# Multiple regulators control the biosynthesis of brasilicardin in *Nocardia terpenica*

**DOI:** 10.1007/s00253-025-13485-3

**Published:** 2025-06-24

**Authors:** Marcin Wolański, Michał Krawiec, Kay Nieselt, Tobias Schwarz, Dilek Dere, Bernhard Krismer, Carolina Cano-Prieto, Harald Gross, Jolanta Zakrzewska-Czerwińska

**Affiliations:** 1 Faculty of Biotechnology, University of Wrocław, Wrocław, Poland; 2 Institute for Bioinformatics and Medical Informatics, University of Tübingen, Tübingen, Germany; 3 Infection Biology Unit, Interfaculty Institute for Microbiology and Infection Medicine Tübingen, University of Tübingen, Tübingen, Germany; 4 Department of Pharmaceutical Biology, Pharmaceutical Institute, University of Tübingen, Tübingen, Germany

**Keywords:** Immunosuppressant, Actinomycetes, Transcriptional factors, Gene expression, Secondary metabolite gene clusters

## Abstract

**Abstract:**

Brasilicardin A, BraA, is a secondary metabolite produced by the bacterium *Nocardia terpenica*, and a promising drug due to its potent immunosuppressive activity and low cytotoxicity. Currently, a semisynthetic approach confers the production of a complete compound but suffers from limited heterologous biosynthesis of BraA intermediates used in the chemical semi-synthesis steps leading to only lab-scale quantities of the compound. A better understanding of the gene expression regulatory pathways involved within the brasilicardin biosynthetic gene cluster, Bra-BGC, is a prerequisite to improving production titers further. However, the transcriptional regulation of the Bra-BGC has only been superficially analyzed, till now. In this study, we comprehensively analyze the functions of several unstudied transcriptional regulators, KstR, SdpR, and OmpR, encoded within the close vicinity of the Bra-BGC, and delve into the role of the previously described cluster-situated activator Bra12. We present that Bra12 and the novel regulator SdpR bind several DNA sequences located in the promoter regions of the genes essential for BraA biosynthesis. Subsequently, we demonstrate the complex regulatory network through which both regulators can control the activity of those gene promoters and thus gene expression in Bra-BGC. Furthermore, using the heterologous producer strain *Amycolatopsis japonicum*, we present that Bra12 and SdpR regulators play opposite roles in brasilicardin congener biosynthesis. Finally, we propose a comprehensive model of multilevel gene expression regulation in Bra-BGC and propose the roles of locally encoded transcriptional regulators.

**Key points:**

• *Multiple regulators bind within the brasilicardin gene cluster.*

• *Bra12 and SdpR are key regulators of brasilicardin biosynthesis.*

• *The bra0 - 1 intergenic region is likely a key regulatory “hot-spot.”*

**Supplementary Information:**

The online version contains supplementary material available at 10.1007/s00253-025-13485-3.

## Introduction

Bacteria of the phylum Actinomycetota (formerly Actinobacteria) (Oren and Garrity 2021) are prolific producers of clinically valuable secondary metabolites (SMs). These include a variety of compounds exhibiting diverse biological activities, e.g., antimicrobial, anticancer, and immunosuppressive. Among Actinomycetota, the genus *Streptomyces* is the main source of commercially available SMs, producing approximately two-thirds of antibiotics of natural origin. A tremendous increase in the number of sequenced bacterial genomes and the development of bioinformatics tools in recent years largely facilitated the identification of novel secondary metabolite gene clusters (SM-BGCs)—this has revealed that the underexplored and so-called rare Actinomycetota, e.g., *Amycolatopsis, Catenulispora*, and *Nocardia*, can also represent precious sources of valuable metabolites (Ding et al. 2019; Engelbrecht et al. 2021; Gavriilidou et al. 2022). However, the discovery of novel compounds is hampered as most of the SM-BGCs are not expressed at all or only at a very low level, making their putative products difficult to detect and study under laboratory conditions. The genome mining strategy followed by genetic engineering tools and heterologous host expression allows for the unraveling of this treasure trove by enabling the activation of silent SM-BGCs (Gross 2007; Rutledge and Challis 2015; Ding et al. 2019; Lee et al. 2020; Bauman et al. 2021). One means of awakening silent SM-BGCs includes engineering transcriptional regulators (TRs) responsible for controlling gene expression in those clusters. This usually involves overexpression of positive and/or elimination of negative TRs. The choice of strategy often relies on a multistep experimental analysis of regulatory protein function. That includes identifying TR target promoters within the gene cluster and TR binding sites, and deciphering the TR mode of action, including putative interaction of the TR with small ligand compounds or/and other protein regulators, or/and its posttranslational modifications.

Brasilicardin A (BraA) is an immunosuppressive compound produced naturally by the human pathogenic bacterium *Nocardia terpenica* IFM 0406 (Shigemori et al. 1998). To date, the physiological or ecological role of BraA in its native host remains unknown. However, BraA is considered a promising lead in organ transplantation due to its high immunosuppressive activity and novel mode of action (Usui et al. 2006). Importantly, BraA exhibits lower toxicity than other drugs currently used, such as cyclosporin (Komaki et al. 2000). The biosynthetic production of BraA using the native producer strain is elaborate, since it requires as a biosafety level 2 (BSL- 2) classified strain strict safety measures during the production and workup phase, and in addition, it exhibits low isolation yields. Furthermore, the preparation of this complex natural product by total synthesis (Anada et al. 2017; Yoshimura et al. 2018) has been impressively demonstrated but is economically not feasible and not sustainable. However, a recent semisynthetic strategy that employed chemical modifications of the BraE compound, an intermediate of the BraA biosynthetic pathway obtained through heterologous expression in a *Streptomyces griseus* host, allowed for the economical production of BraA on a gram scale (Botas et al. 2021). Despite these achievements, further improvement of the titer of BraE is desirable, as this may subsequently facilitate the generation of new brasilicardin derivatives (Niman et al. 2023). However, still little is known about the mechanisms that regulate the expression of the genes involved in brasilicardin biosynthesis, making this task challenging.

In *N. terpenica*, the brasilicardin biosynthetic gene cluster (Bra-BGC) involved in the synthesis of the core skeleton and BraA modifications comprises 13 genes (Fig. 2). These genes include *bra0*–*bra11*, which encode enzymes responsible for the biosynthesis of the compound, and *bra12*, which encodes Bra12, a crucial positive TR of the gene cluster. Bra12 is required for the transcription of all Bra-BGC genes, including its gene (Schwarz et al. 2018a). However, the target promoter regions for Bra12 within the Bra-BGC have not been identified so far. Additionally, genes coding for other putative regulators, namely KstR, SdpR, LysRNt, and OmpR, were recently identified in the regions directly flanking Bra-BGC (see Tab. S4). Among them, only LysRNt has been studied to date (Wolański et al. 2021). LysRNt was shown to play a negative role in the biosynthesis of BraA intermediates, possibly by controlling the expression of a significant portion of the genes within the Bra-BGC through LysRNt binding within the promoter regions of the *bra0 - 1* and *bra12* genes and a putative promoter region of the *bra7* gene. Interestingly, that study also showed that the DNA-binding activity of LysRNt is subject to regulation via the intermediates of the BraA biosynthetic pathway.

The presence of multiple putative transcriptional regulators suggests a complex regulation of Bra-BGC expression, which is in addition possibly influenced by external factors. Since these mechanisms remain elusive, we focused our efforts on collectively examining the roles of the unstudied regulatory protein candidates (KstR, SdpR, OmpR) and exploring the function of Bra12. In this study, out of the three putative regulators, we identify SdpR as a new player that can control gene expression within the Bra-BGC. Using electrophoretic mobility shift assay, DNase I footprinting, and reporter assays, we demonstrate the possible interplay between SdpR and Bra12 regulators in controlling the activity of crucial *bra0–bra1* gene promoters.

## Materials and methods

### Bacterial strains, culture conditions

The strains used in this study are listed in Table S1 in the Supplementary Information (SI). For *Escherichia coli* cultures, the conditions, media, and antibiotic concentrations followed commonly used protocols (Sambrook and Russell 2001); for *Amycolatopsis japonicum* cultures, the growth conditions and media were the same as described previously (Schwarz et al. 2018a); for *Nocardia* and *Streptomyces* cultures, the growth conditions are included in experimental procedures described below. The expression of recombinant proteins in *E. coli* hosts was performed as described below in the paragraph on protein purification.

### DNA manipulations, plasmid, and strain construction

For plasmid construction, standard molecular biology procedures and the SLIC method were used (Kieser et al. 2000; Sambrook and Russell 2001; Li and Elledge 2007). Purification of plasmids and PCR products was performed using commercially available kits (Thermo Fisher Scientific and A&A Biotechnology). Transformation of *E. coli* cells with the linear or circular DNA constructs was conducted as described previously (Kieser et al. 2000; Sambrook and Russell 2001). Plasmid constructs were verified by restriction digestion and/or DNA sequencing. To introduce plasmids into *Streptomyces* and *A. japonicum* hosts, the original (Kieser et al. 2000) and the modified intergeneric conjugation procedure was used (Schwarz et al. 2018a). Plasmid and strain construction strategies are described in the SI. Plasmids, fosmids, and oligonucleotides (supplied by Merck) used in the study are listed in Tables S1 and S2; enzymes were purchased from Thermo Fisher Scientific and New England Biolabs.

### *Nocardia* cultures for RNA isolation, RNA-seq library preparation, and sequencing

Seed cultures of *N. terpenica* IFM 0406 were grown from spore stocks in GPM medium (pH 7.0 ± 0.2) for 39.25 h at 37 °C and 150 rpm. Subsequently, four 300-mL Erlenmeyer flasks (A, B, C, and D) containing 100 mL of GPM medium were inoculated with 300 µL of seed culture and incubated for 33 and 48 h, respectively, at 37 °C with shaking at 150 rpm. These two time points were chosen, since at 33 h, no biosynthesis of brasilicardin A was detected, but at the same time, the *Nocardia terpenica* IFM 0406 cell culture had entered the exponential growth phase, and at 48 h of growth, the biosynthesis of brasilicardin A was detectable.

RNA was then isolated for the four samples, which were named R1 - 33 h, R2 - 33 h, R1 - 48 h and R2 - 48 h. To prepare samples for RNA purification, 20 mL main culture aliquots from the four shake flasks A–D were quickly transferred after 33 h and 48 h, respectively, into 50-mL centrifuge tubes containing 2.2 mL of phenol-ethanol stop solution (one part phenol equilibrated with TRIS/EDTA at pH 8.0 and nine parts of EtOH). Upon mixing by vortexing, each solution was incubated for 5 min on ice and subsequently centrifuged for 15 min at 4500 g and 4 °C. Supernatants were discarded, and the resulting *Nocardia* cell pellets were stored until RNA extraction at − 80 °C. For cell lysis, each pellet was suspended in 4 mL TRIzol and incubated for 5 min at RT. One mL of each TRIzol cell suspension was transferred into 2-mL tubes with 0.5-mL zirconium beads (0.1–0.2 µm in diameter), and cells were disrupted by shaking employing a FastPrep- 24 bead beating instrument (MP Biomedicals) 2 × 30 s at 6 m/s. The cell lysate was kept on ice for 3 min and subsequently mixed with 200 µL chloroform and centrifuged (5 min, 10,000 g at RT). For alcohol-based RNA precipitation, the upper aqueous phase was transferred into a new tube, mixed with 500 µL isopropanol, and incubated for 10 min at − 80 °C. After centrifugation (10 min, 21,000 g, 4 °C), the supernatant was decanted and the resultant pellet was dried. The RNA pellet was dissolved in 100 µL RNase-free water and purified, employing a NucleoSpin® RNA Clean-up kit according to the manufacturer’s protocol. The four extracted RNA samples (R1 - 33 h, R2 - 33 h, R1 - 48 h, and R2 - 48 h) were finally obtained each in 50 µL RNase-free water, quantified using a NanoDrop 1000 instrument, checked for quality using gel electrophoresis, and stored at − 80 °C.

For the library preparation, the four samples were first treated with T4 polynucleotide kinase. The RNA samples were then split into two halves and one half was treated with Terminator™ 5′-phosphate-dependent Exonuclease (+ TEX), and the other half was left untreated (− TEX), as described previously (Sharma et al. 2010). TEX is an enzyme specific for already processed or cleaved transcripts. To generate mainly sequencing reads of the 5′-end of the transcripts, the samples were not fragmented before cDNA synthesis. The cDNA libraries were constructed by Vertis Biotechnologie AG, Germany. For cDNA synthesis, the RNA samples were poly(A)-tailed to the 3′-end using poly(A) polymerase. Then, the 5′-triphosphate residues were removed using RNA 5′-pyrophosphatase. Accordingly, an RNA adapter was ligated to the 5′-monophosphate residue of the RNA. First-strand cDNA synthesis was performed using an oligo(dT)-adapter primer and the M-MLV reverse transcriptase. The resultant cDNAs were PCR-amplified to approximately 10–20 ng/µL using DNA polymerase. Finally, the cDNAs were purified using an Agencourt AMPure XP kit and analyzed via capillary electrophoresis.

For Illumina NextSeq sequencing, the eight cDNA samples (R1 - 33 h-TEX^+^, R2 - 33 h-TEX^+^, R1 - 33 h-TEX^−^, R2 - 33 h-TEX^−^, R1 - 48 h-TEX^+^, R2 - 48 h-TEX^+^, R1 - 48 h-TEX^−^, R2 - 48 h-TEX^−^) were pooled in about equimolar amounts. Subsequently, the cDNA pool was eluted from a preparative agarose gel in the 200–500 bp size range. The primers used for PCR amplification were designed for TruSeq sequencing according to the instructions of Illumina. The cDNA pool was single-end sequenced on an Illumina NextSeq 500 system using a read length of 1 × 75 bp.

### RNA-seq data analysis

For the 5′ enriched sequencing data analysis, the raw reads were first processed with READemption (Förstner et al. 2014), followed by TSSpredator. READemption is an automated RNA-Seq processing pipeline with the purpose of processing differential RNA-Seq (dRNA-Seq) data for the identification of transcriptional start sites in bacteria. READemption consists of several subcommands, for the analysis of the data up to generating the wiggle files that served as input for TSSpredator. In particular, the following subcommands were used: subcommands “create” and “align” using default arguments with the *N. terpenica* IFM 0406 (NCBI RefSeq accession number: NZLWGR00000000.1) for the mapping step. The subcommand “gene_quanti” was used to compute the read quantities based on the respective gff file of the reference genome. Finally, using the subcommand “coverage,” the wiggle files were generated.

Based on the wiggle files computed using READemption, TSSpredator was conducted. TSSpredator is a comparative, fully automated TSS annotation tool for precise and fast generation of genome-wide TSS maps from dRNA-seq data (Dugar et al. 2013). Before the comparative evaluation, the differential RNA-seq expression wiggle data were further normalized using an enrichment normalization percentile approach. The transcription start sites were computed using the mode “comparison of different conditions” in TSSpredator comparing the two different time points of the growth curve (33 and 48 h). The TSS prediction approach was applied at both times to the two biological replicates, respectively, using the default parameter settings of TSSpredator. All computed TSS were classified with respect to their position in the genome relative to annotated, proteinogenic genes (Sharma et al. 2010). For each TSS, it was calculated and investigated whether the site was a primary or secondary transcription start site of a gene, whether an internal TSS was proved, an antisense TSS, or whether the site could not be assigned to any of these classes of annotated genes (orphan TSS).

Raw sequencing reads in FASTQ format as well as the final TSS master table (Tab. S6) computed as described above are available via Gene Expression Omnibus under accession number GSE271981 (https://www.ncbi.nlm.nih.gov/geo/query/acc.cgi?acc=GSE271981).

### RT-qPCR

For details on RNA isolation and RT-qPCR analysis of *A. japonicum* strains, see the SI.

### Protein purification

The recombinant KstR-His, SdpR-His, Bra12-His, and OmpR-His proteins were purified using approaches described earlier in detail for LysRNtHis_6_ (Wolański et al. 2021). Briefly, the *E. coli* Rosetta™ 2(DE3) strains harboring corresponding regulatory genes cloned in pET- 21a(+) expression plasmid were used to express C-terminally His-tagged proteins. The proteins were purified on the Äkta Start system (GE Healthcare) using metal affinity chromatography resins (Talon column). The protein purity was assessed using sodium dodecyl sulfate–polyacrylamide gel electrophoresis (SDS-PAGE) (Laemmli 1970) and staining (PageBlue™ solution from Thermo Fisher Scientific).

### Protein-DNA interaction studies

Electrophoretic mobility shift assay (EMSA) and DNase I footprinting were used to investigate interactions between recombinant proteins and the DNA fragments representing either the selected regions on the BcaAB01 fosmid encompassing the gene cluster or the entire fosmid. For EMSA, a standard approach described earlier (Wolański et al. 2021) with modifications was applied—for details, see the SI. DNase I footprinting was conducted as described in the previous study (Wolański et al. 2016).

### Luciferase reporter assay

The activities of the gene promoters were measured in the *Streptomyces coelicolor* M1154 host using bacterial luciferase reporter assay, as described previously (Płachetka et al. 2021). Briefly, the spores of the *Streptomyces* strains harboring corresponding pFLUX vectors, bcaAB01 fosmid (or its derivatives), or pUWL vector (or its derivatives) (Tab. S2) were applied onto the solid DNA medium previously poured into the wells of white opaque 96-well plates (OptiPlate- 96, PerkinElmer). The wells were inoculated with 7.5 µL of activated (10 min, 50 °C) spore suspensions of OD_600_ = 3.3. Three biological replicates (three independent clones per strain) and four technical replicates per clone were used in the measurements. The plates were incubated in the inverted orientation at 30 °C, and the luminescence readings were taken using an EnVision 2105 multimode plate reader (Perkin Elmer).

### Detection of brasilicardin congeners

To assess the levels of brasilicardins production in *A. japonicum* strains, the procedure described in the previous study was applied (Schwarz et al. 2018a). Briefly, a preculture grown in tryptic soy broth medium (TSB) for 48 h was subsequently diluted with SM17 medium at a ratio of 1:100 and cultivated for another 72 h. The centrifuged supernatants were used directly for HPLC/MS analysis. The UV intensities obtained for each brasilicardin congener (BraC, BraC aglycon, BraD, and BraD aglycon) were summed up to calculate total brasilicardin production.

### Other bioinformatics tools, nucleotide, and amino acid sequences

The web addresses for bioinformatics tools and the links to sequence resources used in the study are listed in the SI (Tab. S7 and Supplementary Materials and Methods). Briefly, the following tools were used: protein parameters—ProtParam (Gasteiger et al. 2005); identification of protein domains—SMART (normal mode) (Schultz et al. 1998; Letunic and Bork 2018) and CD search tool (default parameters) (Marchler-Bauer et al. 2017); searches for protein homologs—BLASTp (protein–protein blast); in silico prediction of protein DNA binding sites—MEME tool of MEME suite package (Bailey et al. 2009) (search parameters were as follows: classic mode; maximum 3 different sequence motifs; occurrence 0 or 1 per sequence); generation of sequence logos—WebLogo (Crooks et al. 2004). The following sequence data are available online: *Nocardia terpenica* IFM 0406 genome sequence—EMBL/GenBank, accession number LWGR00000000.1 (Buchmann et al. 2016); the Bra-BGC sequence—GenBank, accession number MT247069. CorelDRAW X5 was used to create the figures.

## Results

Several previous attempts to achieve a high production yield of BraA in native and heterologous host systems turned out to be highly inefficient, most likely due to the low transcriptional activity of the Bra-BGC (Schwarz et al. 2018b, 2018a; Wolański et al. 2021). To verify this assumption, we examined global gene transcription in the native host *N. terpenica* IFM 0406 strain using RNA-seq. This analysis revealed that the expression of the brasilicardin gene cluster (average RPKM 4.5 and 4.2 at 33 h and 48 h, respectively) and many other secondary metabolite gene clusters, for terpenibactins (Chen et al. 2020), ectoin or, 2-methylisoborneol biosynthesis (BGCs 1.10, 1.4, and 1.6, respectively), exhibited significantly lower expression levels than primary metabolism genes (e.g., for ribosomal proteins, 16S rRNA or sigma factors) (Tabs. S3A and B).

**Fig. 1:**
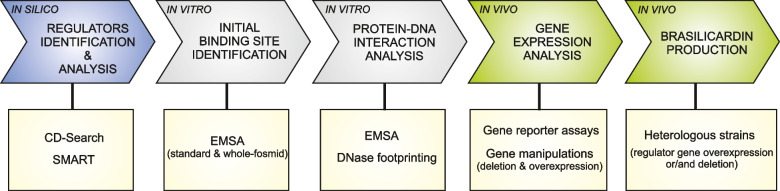
Overview of the experimental strategies used in this study. Schematic representation of the workflow used to decipher functions of transcriptional regulators identified within the Bra-BGC. The milestones are listed in the color-coded arrows of the panel, and the corresponding experimental techniques are shown below in rectangular boxes

Since our goal was to fully understand the mechanism regulating gene expression within the Bra-BGC, we embarked on a detailed study of the impacts of the transcriptional regulators (TRs) identified in silico within the Bra-BGC region (Wolański et al. 2021). In this study, we focused on Bra12 and three other putative regulators, namely KstR, SdpR, and OmpR, which have not been studied yet. To decipher their roles, we employed comprehensive experimental approaches following the general strategy presented in Fig. 1.

### Local regulators bind targets within Bra-BGC

#### In silico analysis identifies putative novel regulators of the Bra-BGC

**Fig. 2:**
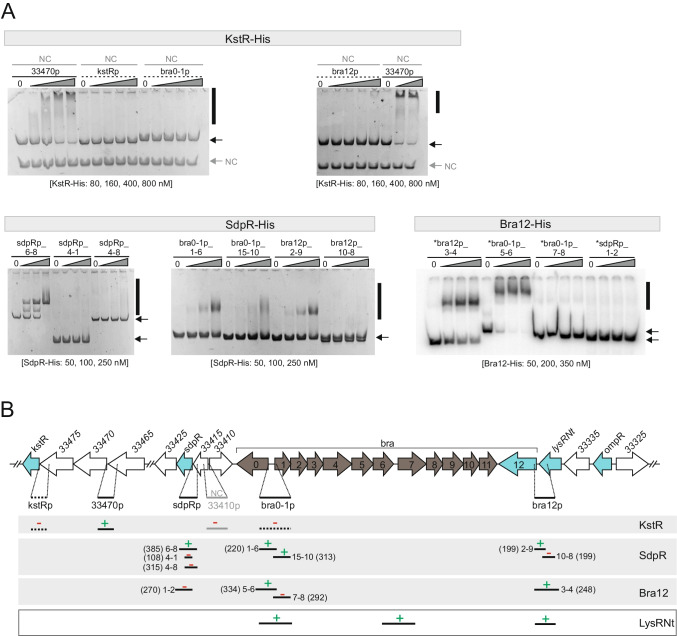
Identification of binding sites of regulatory proteins within the Bra-BGC. **A** EMSA. The recombinant proteins (Bra12-His, SdpR-His, KstR-His) at varying concentrations (indicated below the gels) were incubated with the same amounts of PCR-amplified DNA fragments comprising selected promoter regions (see Figs. S2–5). Unlabeled DNA was used for assays with KstR and SdpR proteins, and ^32^P-labeled DNA (marked with an asterisk) for assays with Bra12. The DNA fragments indicated with the solid lines represent regions identified in the preliminary EMSA (Figs. S2–5); those in gray indicate the internal negative control (NC) (33410p fragment). Black vertical bars along the gels indicate observed protein-DNA complexes; black and gray arrows indicate unbound DNA fragments and the internal NC. **B** EMSA—graphical summary. The DNA fragments analyzed are indicated by short horizontal bars. Numbers next to those bars correspond to primers used in the PCR reactions (Tab. S2); numbers in brackets represent fragment sizes (bp). The presence and absence of protein-DNA interaction are indicated by “** + **” (in green) and “** − **“ (in red) symbols, respectively. The very bottom panel presents previously identified binding locations for the LysRNt protein (Wolański et al., 2021)

To gain a first insight into the roles and confirm putative DNA-binding properties of the TRs identified in the Bra-BGC, we analyzed their amino acid sequences for the presence of functional protein domains using SMART and conserved domain-search (CD-search) tools. The analyzed proteins, including the previously studied LysRNt, comprise N-terminal DNA-binding domains (DBDs), except OmpR which contains the DBD at its C-terminus (Tab. S4 and Fig. S1A). The helix-turn-helix (HTH) (KstR and LysRNt) and winged-helix-turn-helix (wHTH) (SdpR, Bra12, OmpR) motifs are assumed to confer these DNA-binding functions. The C-terminal (N-terminal in case of OmpR) portions of those proteins encode putative regulatory domains (RD) involved presumably in signal-receiving functions, for instance, ligand binding (e.g., ADP in Bra12). The analyzed TRs are small proteins (MW < 35 kDa), except for Bra12 which exhibits a higher MW (66.1 kDa). The BLASTp search against nonredundant protein sequences in the NCBI database indicated that all TRs are relatively widespread in bacteria and, in the case of KstR, also in Archaea (Tab. S4). A search against *N. terpenica* IFM0406 protein sequences indicated that each of the regulators has at least one putative paralogue protein in the native host (Tabs. S5A–E). The relatively large, in comparison to KstR and SdpR, number of paralogues in the case of the Bra12, OmpR, and LysRNt (1–2 vs 14–27, respectively) suggests that the paralogs of the latter group of proteins are likely to cover various cellular processes and respond to a variety of environmental stimuli. Indeed, the Bra12 homolog of the AfsR/SARP family, AfsR, is a global regulator activated by phosphorylation and involved in complex regulatory networks in *Streptomyces* (Tanaka et al. 2007). Consistently, the OmpR family of regulators frequently comprises two-component systems involved in the transduction of environmental signals (Itou and Tanaka 2001; Li et al. 2014). Importantly, *ompR* of Bra-BGC is accompanied by a divergently orientated gene (AWN90_RS33325) (Fig. 2) encoding a putative receptor histidine kinase that might be responsible for the phosphorylation of the OmpR regulator. The homologs of KstR and SdpR regulators are less extensively studied. The KstR belongs to a large family of TetR proteins that play roles in a variety of cellular processes (Ramos et al. 2005), including nitrogen metabolism (Jakoby et al. 2000; Loh et al. 2006). Interestingly, the analysis of the gene organization indicates that *kstR* forms a presumable operon with two downstream genes *33,470*–*33475*, coding for enzymes related to nitrogen (amino acid) metabolism pathways. The SdpR putative regulator belongs to a large family of metalloregulators, widespread in bacteria, of which some act as metal sensing repressors involved in metal resistance processes, e.g., ArsR in *E. coli*, SmtB in *Synechococcus*, and CmtT in *Mycobacterium* (Erbe et al. 1995; Xu et al. 1996; Wang et al. 2005).

#### Regulatory proteins bind to several promoter regions within the Bra-BGC

To identify the genes putatively controlled by the regulators, we sought the regulatory protein binding sites within the Bra-BGC using an in vitro approach based on electrophoretic mobility shift assays (EMSAs). Proteins used in these assays were expressed as C-terminal fusions with Hisx6-tag (Fig. S1B) and purified as previously described (Wolański et al. 2021). In the initial screen for the TR binding sites, we used as DNA bait the restriction-digested fosmid bcaAB01 that comprises the Bra-BGC and the flanking regions of the cluster. This approach led to the identification of several DNA regions bound by the KstR, SdpR, and Bra12 proteins over bcaAB01 (Figs. S2-4). However, the screen turned out to be unsuccessful for OmpR as we did not observe an interaction of this protein with any bcaAB01 fosmid DNA fragment (Fig. S5). This resulted in the exclusion of the protein from further analysis.

Next, to more precisely examine if the other regulators bind to regions containing gene promoters (designated using RNA-seq results, Fig. S11), we performed a set of EMSA experiments using promoter regions located within the fished-out DNA fragments. These included promoters of the investigated regulatory genes *sdpR*, *bra12*, and *kstR* (in the latter case, the *33,470* gene promoter was used as the *33,470*–*33475*-*kstR* form putative gene operon), and essential biosynthetic genes *bra0*, *bra1* (sharing a common divergent intergenic promoter region), and *bra7* (forming the presumed *bra7*-*bra11* sub-operon) (Wolański et al. 2021). The EMSAs revealed the interaction of both SdpR and Bra12 regulators with the fragments comprising the divergent promoter region of *bra0*–*bra1* genes (bra0 - 1p) and the promoter region of *bra12* gene (bra12p) (Fig. 2). In addition, SdpR exhibited binding to its gene promoter region (sdpRp). Noteworthy, the pattern observed for the SdpR-sdpRp interaction suggested the presence of multiple SdpR binding sites within the investigated promoter region. The KstR protein showed an interaction with the 33470p fragment, which contained a presumed promoter region of the putative *33,470*–*33475*-*kstR* gene operon. However, since *33,470*–*33475* genes presumably do not play a role in BraA biosynthesis, as inferred from previous studies (Schwarz et al. 2018a), we excluded KstR from further analysis at this stage. Importantly, the putative promoter of *bra7* was not bound by either SdpR or KstR (Fig. S6). This region was previously demonstrated as the target for another Bra-BGC regulator, LysRNt (Wolański et al. 2021).

In sum, the in vitro-based protein-DNA interaction studies identified SdpR and Bra12, as the most promising regulators for further analyses. Both SdpR and Bra12 proteins bind to the promoter regions of *bra0–bra1* and *bra12* genes crucial for brasilicardin biosynthesis. The interaction of SdpR with the *sdpR* gene promoter region suggests that SdpR presumably regulates the transcription of its own gene.

### SdpR and Bra12 regulate the activities of gene promoters within the Bra-BGC

#### SdpR and Bra12 bind specific sequences within the promoter regions

EMSA analyses revealed *sdpR*, *bra0 - 1*, and *bra12* gene promoter regions as the targets for SdpR and Bra12 regulators in the Bra-BGC. To gain more insight into protein-DNA interactions, we next aimed to identify the binding sites and to determine the binding sequences for both the TRs.

**Fig. 3:**
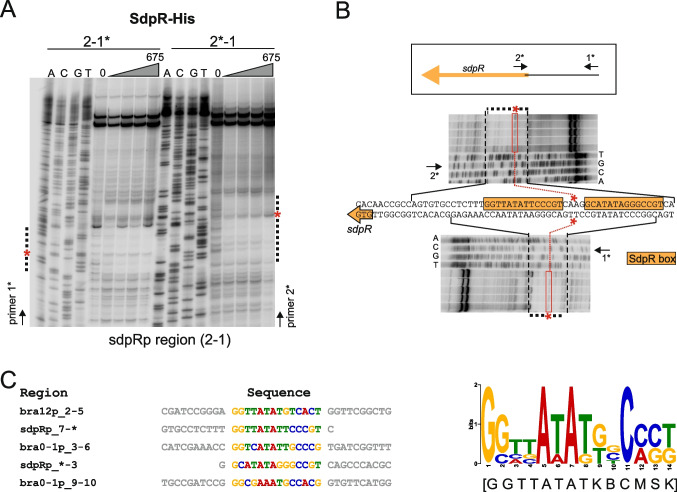
Determination of the SdpR binding sequence. **A** DNase I footprinting. The SdpR-His protein (25, 75, 225, and 675 nM final concentrations) was incubated with the ^32^P-labeled sdpRp_1 - 2 DNA fragment (270 bp) (marked with the asterisk), followed by DNase I treatment. The DNase I-protected or DNase I hypersensitive regions are marked with vertical dotted lines and red asterisks (*), respectively. The sequencing reactions are designated with A, C, G, and T. **B** The detailed analysis of DNase I-protected sequences and the locations of SdpR boxes. **C** (Left) The alignment of the sequences containing the predicted SdpR boxes. The MEME in silico tool was used to predict SdpR sequences within the DNA fragments selected by EMSA (see Fig. S7). (Right) The SdpR box sequence logo and consensus sequence (below in square brackets) were obtained using MEME

First, we examined SdpR binding sites using the DNase I footprinting and EMSA. The footprinting assay, carried out in the presence of the DNA fragment that included the promoter of the *sdpR* gene (sdpRp_1 - 2), revealed two DNase I-protected regions (Fig. 3A and B), indicating the presence of two SdpR binding sites. This finding was in line with the previous assumption (Fig. 2A) and confirmed using shift assays with the subfragments of the *sdpRp* region (Fig. S7A). Interestingly, in the DNase I footprinting, we also observed the presence of the DNA region prone to DNase I digestion (marked with asterisks in Fig. 3A and B). This DNase I hypersensitive site might result from protein-induced DNA bending occurring when a protein interacts with a DNA region containing closely located binding sites. Importantly, we could not observe any DNase I-protected regions while investigating the interactions of SdpR with the fragments that encompass *bra0 - 1* and *bra12* promoters (data not shown). This can be explained by a much weaker interaction of SdpR with those two fragments (weak interaction at ~ 100 nM of SdpR) than with the fragment comprising the *sdpRp* region (strong binding at 50 nM of SdpR) as observed earlier in EMSA experiments (Fig. 2A). Thus, to narrow down the DNA sequences that contain SdpR binding sites within these regions, we used standard and competition EMSA (Fig. S7B and C). These results exhibited binding of the SdpR regulator to two distinct locations within *bra0 - 1p* and one location within the *bra12p* region. The sequences of the identified fragments and the two *sdpRp* subregions comprising SdpR binding sites were further subjected to an in silico search for common sequence motifs using the MEME tool (Fig. 3C, left panel, and Fig. S7). This led to the discovery of a 14-nucleotide SdpR binding sequence, hereafter referred to as the SdpR box (Fig. 3C, right panel). The double SdpR boxes have been identified within the *sdpRp* and *bra0 - 1p* regions and a single SdpR box within the *bra12* promoter region. The boxes exhibit a unique arrangement and locations with respect to the corresponding transcriptional start sites (TSSs) in the corresponding gene promoter regions (Fig. S7, Fig. S8, and Tab. S6).

**Fig. 4:**
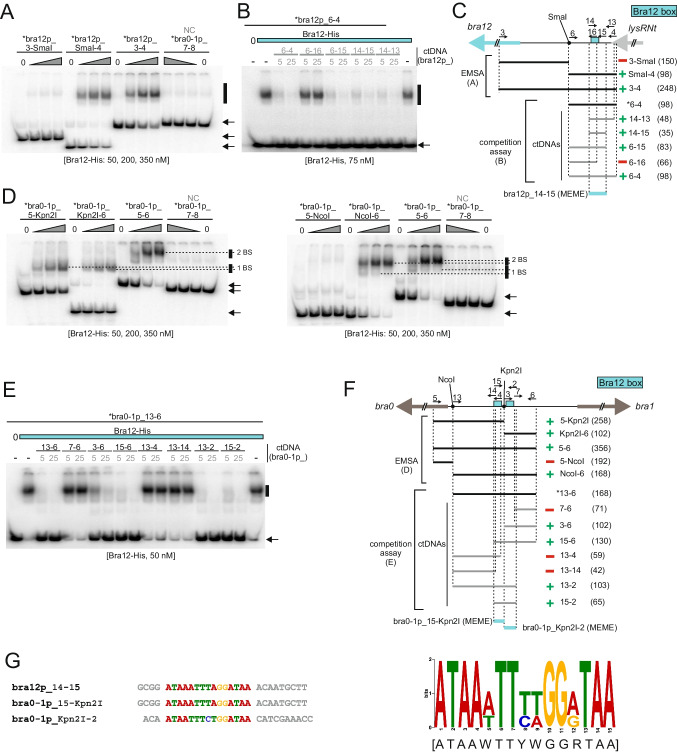
Determination of the Bra12 binding sequence. **A–C** and **D–F** The identification of Bra12 binding sites within *bra12* and *bra0 - 1* promoter regions, respectively. **A** and **D** EMSA. The Bra12-His protein (concentrations shown below the gels) was incubated with the sets of *bra12p* or *bra0 - 1p* regions sub-fragments or a negative control (NC) fragment (bra0 - 1p_7 - 8, see also Fig. 2B). “1 BS” and “2 BS” (**D**) refer to protein-DNA complexes formed by the DNA comprising 1 and 2 Bra12 binding sites, respectively. **B** and **E** Competition EMSA. Bra12-His (75 nM, blue bar) was incubated with radioactively labeled bra12p_4 - 6 or bra0 - 1p_13 - 6 fragments (~ 0.2 nM) and two sets of “cold-target DNA” competitors (ctDNA) (5 or 25 nM) (PCR-amplified DNA indicated in gray font and lines). Black vertical bars and black arrows (**A**, **B** and **D**, **E**) indicate protein-DNA complexes and unbound DNA, respectively. **C** and **F** The summaries of panels **A**, **B** and **D**, **E**, respectively. Radiolabeled primers and DNA fragments are indicated with asterisks (*). The green symbols “** + **” indicate Bra12-His interactions of Bra12-His with corresponding DNA fragments (EMSA) or the ability of the corresponding “cold-target DNA” fragment to outperform the radiolabeled probe used (competition EMSA); the red symbol “** − **“ represents either the lack of protein-DNA interaction (EMSA) or the lack of the ability of the “cold-target DNA” fragment to outperform the ^32^P-labeled DNA fragment (competition EMSA). The numbers next to the bars represent the primers used in the PCR reactions (Tab. S2); numbers in brackets represent fragment sizes (bp). **G** (Left) Alignment of sequences containing the predicted Bra12 boxes. The MEME in silico tool was used to predict Bra12 sequences within EMSA-selected DNA fragments (fragments marked with blue bars in panels **C** and **F**). (Right) Bra12 box sequence logo and the consensus sequence (below in square brackets) were obtained using MEME

Next, we aimed to identify Bra12 regulator binding sites within the *bra0 - 1p* and *bra12p* regions. As no DNase I protection patterns were observed in the footprinting assays (data not shown), we applied as previously an EMSA (including competition assay) approach. In the initial EMSA experiments, we narrowed down the DNA fragments comprising Bra12 binding sites in both promoter regions (Fig. 4A and D). Moreover, the results also suggested the existence of single- and double-binding sites within the *bra12p* and *bra0 - 1p* regions, respectively. By further narrowing down the sequences containing Bra12 regulator binding sites using competition EMSA (Fig. 4B, C, E, and F) and subsequently in silico searches using MEME, we were able to identify the 15-nucleotide Bra12 binding sequence, hereafter called the Bra12 box (Fig. 4G, right panel). All the identified boxes are located upstream of the previously designated TSSs (Fig. S11) in those gene promoters (Fig. 7), suggesting that the protein acts as a positive transcriptional regulator for the corresponding genes. The two Bra12 boxes located in *bra12p* and the left portion of *bra0 - 1p* share identical sequences, while the third Bra12 box, located in the right portion of the *bra0 - 1p* region, differs in three positions from the former sites (Fig. 4G). Intriguingly, in the competition shift assay employing the radiolabeled DNA fragments comprising *bra12p* and *bra0 - 1p* regions (Fig. 4B–E), we did not observe disruption of the corresponding protein-DNA complexes in the presence of the respective bra12p_6 - 16 and bra0 - 1p_13 - 4 ctDNA fragments, containing the sequences overlapping the corresponding Bra12 boxes in those regions. However, we observed full out-competition of the Bra12 protein binding to those radiolabeled fragments when using longer ctDNA fragments (e.g., bra12p_6 - 15, bra0 - 1p_13 - 2) which were extended by a few or several nucleotides beyond the Bra12 boxes. These findings suggest an inefficient binding of the Bra12 protein to the sites located close to the ends of linear DNA fragments, and the need for the presence of flanking nucleotides necessary to stabilize those protein-DNA interactions.

In addition, due to the presence of non-overlapping Bra12 and SdpR boxes within the intergenic *bra0 - 1p* region, we also attempted to analyze the possibility of simultaneous binding of these proteins to the intergenic region. The EMSA experiments with a subfragment of *bra0 - 1p* (bra0 - 1p_5 - 6) comprising both Bra12 boxes and a proximally located single SdpR box revealed simultaneous and non-competitive binding of Bra12 and SdpR proteins to *bra0 - 1p* (Fig. S10A) and confirmed the relative locations of the Bra12 and SdpR boxes within that intergenic region (Fig. S10B).

Concluding, the abovementioned in vitro experiments allowed us to determine the SdpR and Bra12 binding sites within *sdpR*, *bra0 - 1*, and *bra12* gene promoter regions. Given their locations relative to the gene TSSs in those promoter regions (Fig. 7), positive and complex regulatory functions in the transcriptional control for Bra12 and SdpR regulators have been proposed.

#### Impact of SdpR and Bra12 on gene promoter activity

Protein-DNA interaction analyses allowed the precise localization of the SdpR and Bra12 regulator binding sites within the promoters of the genes crucial for brasilicardin biosynthesis (*bra0*, *bra1*, and *bra12*). To investigate their impacts on target gene promoters, we applied a bacterial luciferase reporter assay. The well-established and easily genetically tractable *S. coelicolor* strain M1154 was used. The strains comprised either the wild-type bcaAB01 fosmid or gene deletion versions of that fosmid (bcaAB01_$\Delta$*sdpR* and bcaAB01_$\Delta$*bra12*) together with the appropriate reporter pFLUX plasmids containing luciferase encoding gene operon *luxCDABE* (Craney et al. 2007) under the control of a studied gene promoter.

**Fig. 5:**
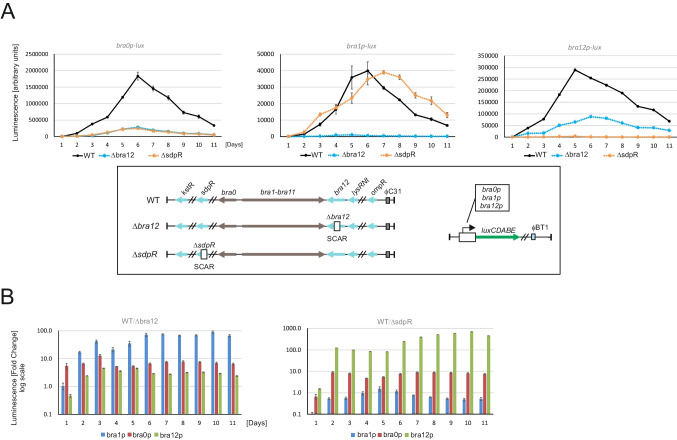
Impact of SdpR and Bra12 regulators on the activities of gene promoters in the Bra-BGC. Luciferase assays. **A** Measurements were conducted using heterologous strains of *S. coelicolor* M1154 that harbor either a *sdpR* or a *bra12* gene deletion fosmid (bcaAB01_$\Delta$sdpR or bcaAB01_$\Delta$ bra12, respectively), and the corresponding gene promoters (*bra0p*, *bra1p*, and *bra12p*) delivered onto pFLUX integrative plasmids. The luminescence readings were normalized against the OD_600_ of the corresponding cultures grown on a solid DNA medium for 11 days. The frame at the bottom depicts the fosmids and plasmids used to create corresponding reporter strains. **B** The fold changes of promoter activities. The graphs were created using averaged luminescence values obtained for corresponding time points

The time-course analyses for the reporter strains showed a negative impact of the *bra12* gene deletion on the activities of the *bra0* and *bra1* gene promoters, as well as its own gene promoter, *bra12p* (Fig. 5). The deletion of *bra12* most significantly affected *bra1p* for which up to a 100-fold reduction in luminescence levels was observed in comparison to the wild-type strain (Fig. 5B). A less pronounced effect of *bra12* gene deletion was observed in the case of the *bra0p* and *bra12p* (up to a tenfold decrease in both cases). The negative impact on gene promoter activities was also observed in the case of *sdpR* gene deletion strains comprising *bra0p* or *bra12p* (Fig. 5), manifested by 10- and 100-fold reductions, respectively (Fig. 5B). The essential role of SdpR for *bra12p* activity was also observed in the reporter strain comprising the *sdpR* gene under the constitutive promoter ermE* (Fig. S9). Interestingly, while only minor differences in luminescence levels of the *bra1p* region between $\Delta$*sdpR* and WT strains were observed, the deletion of *sdpR* exerted a small-time shift in the luminescence activity peak (Fig. 5A, middle panel).

In sum, the gene expression analyses using reporter strains indicated the impacts of both Bra12 and SdpR regulators on the activities of the promoters controlling the genes essential for brasilicardin biosynthesis. The absence of the Bra12 regulator exerts a negative impact on the activities of *bra0*, *bra1*, and *bra12* gene promoters, suggesting that Bra12 acts as a positive transcriptional regulator. The absence of SdpR negatively impacts the activities of the *bra0* and *bra12* gene promoters and exerts a negligible impact on the *bra1* promoter, indicating that the protein presumably plays a complex overall role in gene expression.

### SdpR and Bra12 impact brasilicardin production

To study the influence of regulatory proteins on biosynthesis, we evaluated the production levels of brasilicardin congeners in liquid cultures of several mutant strains. Due to the low genetic tractability of the *N. terpenica* strain, we used the previously established heterologous host *Amycolatopsis japonicum*, also belonging to Actinomycetota (Schwarz et al. 2018a, 2018b). To examine the impact of a lack of regulators, we individually introduced to the host strain the bcaAB01 fosmids comprising *sdpR* or *bra12* gene deletions (bcaAB01_$\Delta$sdpR and bcaAB01_$\Delta$bra12) (Tab. S1). To study the overexpression of the regulatory genes, we cloned the *sdpR* gene into the integrative plasmid pIJ10257 under the constitutive ermE* promoter (ermE*p) leading to the construction of pIJ_sdpR. Subsequently, the pIJ_sdpR and *bra12* overexpression plasmid pIJ_bra12, generated in the previous study (Schwarz et al. 2018a), were introduced into the *A. japonicum* host strain carrying the pPS1 derivative of bcaAB01 fosmid (Tab. S1). The pPS1 facilitates analyses of the impact of individual regulators as it comprises only a minimal set of genes essential for brasilicardins biosynthesis (*bra0*-*bra12*) and confers production of brasilicardins on levels similar to those of the strain harboring the bcaAB01 fosmid (Schwarz et al. 2018a). To analyze the effects of gene deletions, the corresponding derivatives of the bcaAB01 fosmid were individually transferred to the wild-type *A. japonicum* host. In both the overexpression and deletion strains, the corresponding changes in *bra12* and *sdpR* regulatory gene expression levels were confirmed by qPCR (Fig. S12).

**Fig. 6:**
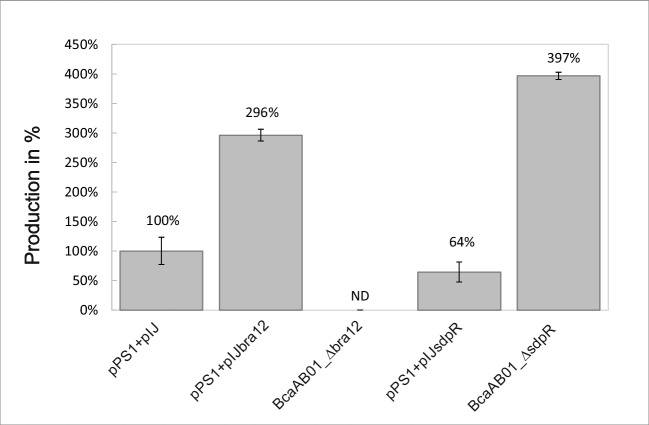
The influence of regulatory proteins Bra12 and SdpR on the biosynthesis of brasilicardin congeners. HPLC/MS. The total production of brasilicardins (sum of BraC, Brac-agl, BraD, BraD-agl) was measured in the supernatants of 72-h liquid cultures of *A. japonicum* strains harboring corresponding constructs (see Tab. S1). The bars represent the averages of three technical replicates obtained for each strain and normalized to *A. japonicum*::pPS1 + pIJ (100%); the standard deviations were calculated using Excel. ND is non-detectable

HPLC/MS analysis of liquid culture extracts of the *bra12* overexpression strain (*A. japonicum*::pPS1 + pIJ_bra12) revealed significantly higher (296%) levels of brasilicardins compared to the control strain harboring the empty plasmid pIJ10257 (*A. japonicum*::pPS1 + pIJ) (100%) (Fig. 6). In support of this result, the *bra12* deletion mutant created in our lab (*A. japonicum*::bcaAB01_$\Delta$bra12) showed no production of brasilicardins, which is also in line with the previous findings (Schwarz et al. 2018a). Conversely, the SdpR overexpression strain (*A. japonicum*::pPS1 + pIJ_sdpR) exhibited a negative effect manifested by a clear drop (64%) in brasilicardin production, while the deletion mutant of this gene (*A. japonicum*::bcaAB01_$\Delta$sdpR) showed pronouncedly higher brasilicardin levels (397%) with the reference to the control strain. To further enhance brasilicardin production, we also attempted to investigate the effect of combining the *sdpR* gene deletion (bcaAB01_$\Delta$sdpR) with *bra12* overexpression (pIJ_*bra12*) in a single strain. However, for unknown reasons, we could not obtain the strain despite several attempts.

To sum up, we confirmed the essential role of the Bra12 protein for brasilicardin biosynthesis using a heterologous host expression system. We also showed that the SdpR regulator negatively affects the production of the corresponding secondary metabolites.

## Discussion

Brasilicardin A is considered a promising immunosuppressive lead structure for organ transplant therapies. The recent findings showed a semisynthetic approach as the effective way to obtain a complete compound in laboratory-scale quantities (Botas et al. 2021). However, further improvements regarding the production yields are limited by the availability of biosynthetically generated brasilicardin intermediates. Amelioration in their production levels seems to be hampered to the greatest extent by the lack of understanding of molecular mechanisms regulating gene expression in the brasilicardin gene cluster, Bra-BGC. The sequence analysis of the gene cluster and its flanking regions revealed the presence of multiple genes encoding putative transcriptional regulators (KstR, SdpR, Bra12, LysRNt, and OmpR) suggesting a complex transcriptional regulatory network (Wolański et al. 2021). However, only the roles of the LysRNt and Bra12 have been investigated to date, in addition, to various extents. While the role of LysRNt regulator in brasilicardin biosynthesis and its presumed mechanism controlling the activity of the protein has been revealed in a recent study (Wolański et al. 2021), the function of Bra12, considered the major positive regulator, has not been explored in detail (Schwarz et al. 2018a). To broaden our understanding of the regulatory dependencies in the Bra-BGC, we embarked on the investigation of the unstudied putative regulators KstR, SdpR, and OmpR and delved into the function of Bra12.

To elucidate the potentials of new regulatory candidates and Bra12, to control gene expression within the Bra-BGC, we first looked for the binding sites for those proteins within the gene cluster. Using the EMSA approach, we demonstrated that KstR, SdpR, and Bra12 proteins were able to interact with several gene promoters located within the regions comprising the Bra-BGC and flanking genes on the fosmid BcaAB01. Nevertheless, we did not detect the binding of OmpR protein to any of the fosmid regions. This led to the subsequent exclusion of this putative regulator from further analyses. Noteworthy, the deletion of the BcaAB01 region comprising the *ompR* gene did not affect brasilicardin biosynthesis in the previous study (Schwarz et al. 2018a), suggesting that the OmpR does not play a function in compound production. The second regulator, KstR, belonging to the TetR family, was also excluded from further investigation, as it only exhibited the potential to regulate the expression of a putative gene operon *33,470*–*33475-kstR*, including its own gene. However, the glutamine-dependent synthetase and peptidyl-arginine deaminase enzymes, encoded by *33,470*–*33475*, usually play roles in nitrogen (amino-acid) metabolism pathways and, according to a previous study, are not related to precursor supply and brasilicardin biosynthesis (Schwarz et al. 2018b). The two other candidate regulators, Bra12 and SdpR, showed greater potential to regulate gene expression in the Bra-BGC as they exhibited binding to the promoters of biosynthetically essential genes *bra0-bra1* and *bra1,* and their own gene promoter regions, suggesting the addition of possible transcriptional autoregulation. Moreover, SdpR exhibited binding to the *bra12* promoter region indicating another level of transcriptional regulation in the gene cluster.

An initial prediction of Bra12 and SdpR functions, based on the locations of their binding sites in relation to the transcriptional start sites (TSS) determined using RNA-seq, led us to propose that Bra12 plays a positive role in gene expression, while the role of SdpR in gene expression cannot clearly be defined. Indeed, quantitative analysis using a reporter gene expression system showed that the strains lacking the *bra12* gene exhibit pronouncedly decreased activity of *bra0*, *bra1,* and *bra12* gene promoters (~ 10-, ~ 100-, and ~ tenfold, respectively), in comparison to the control strains containing the gene. The results correspond to a previous qualitative gene expression study that showed a crucial role of Bra12 in the transcription of the entire set of genes involved in the brasilicardin biosynthesis, namely *bra0* and *bra1–bra11*, and its own *bra12* gene (Schwarz et al. 2018a). We assume that the strongest effect observed for the *bra1* promoter emphasizes the essential role of Bra12 not only in the expression of gene *bra1* but also for the entire *bra1–bra11* biosynthetic gene cluster, as those genes are assumed to form an operon or sub-operon (Wolański et al. 2021). Disruption of *bra12* affects negatively, also, though less pronounced (~ tenfold change), the activity of the promoter of another gene, this time *bra0*, orientated divergently to *bra1*, and sharing within *bra1* a common intergenic region. Noteworthy, *bra0* codes for the dioxygenase involved in decorating brasilicardin congeners with the methoxy group at carbon atom C16. Although, Bra0 does not play an essential role in brasilicardin backbone biosynthesis (Schwarz et al. 2018a), the methoxy group seems to be crucial for the immunosuppressive activity of brasilicardin A (Komatsu et al. 2004, 2005). Altogether, our findings indicate that Bra12 controls the activity of all biosynthetic genes in the gene cluster. We propose that binding Bra12 within the *bra0 - 1* region is required to activate expression of the entire Bra-BGC and suggest that Bra12 alone might exert a major impact on brasilicardin biosynthesis. In addition, the activity of the *bra12* gene promoter shows a positive correlation with the presence of Bra12, indicating autoregulation of its own gene (Fig. 5). The requirement of Bra12 for the transcription of the *bra12* gene was first observed in a previous study (Schwarz et al. 2018a). We demonstrate that this is likely due to direct regulation, as Bra12 binds to the *bra12* gene promoter region. Transcriptional autoregulation was reported for several regulators of the SARP (*Streptomyces* antibiotic regulatory protein) family to which Bra12 belongs (Santamarta et al. 2002; Hoskisson et al. 2006; Novakova et al. 2010; Tsypik et al. 2021). It is also worth mentioning that some of the known AfsR/SARP regulators may undergo phosphorylation, required to enhance their DNA-binding activity. The analysis using the SMART tool identifies an N-terminal DNA binding domain of Bra12 as a Trans_reg_C receiver domain (Pfam PF00486), typical for response regulators of two component phosphorelay transduction systems. In addition, our sequence analysis has identified at least 21 homologs of Bra12 encoded in the *N. terpenica* genome. This suggests that AfsR homologs can likely form a number of regulatory networks responding to various environmental stimuli in this organism. However, putative activation of Bra12 by phosphorylation, and further impact on expression of the Bra-BGC, has not been investigated and remains unknown. Importantly, heterologous production assays confirm the essential role of Bra12 for the biosynthesis of brasilicardins (see further discussion).

**Fig. 7:**
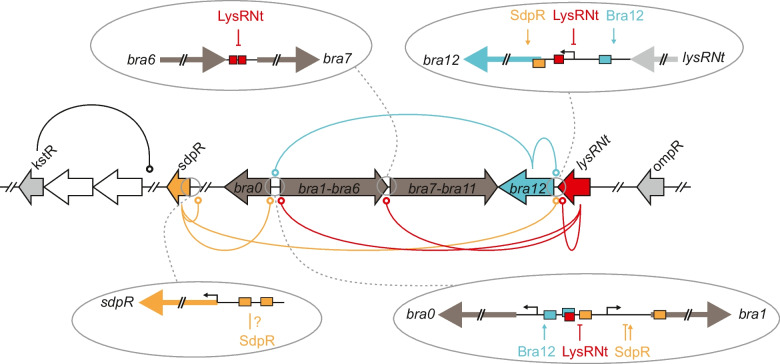
Presumable regulation of the Bra-BGC. The genes encoding regulatory proteins are linked to the identified target gene promoter regions using color-coded solid lines that end up with circles. The blue, orange, and red rectangles within the promoter regions depict the locations of the binding sites of the regulatory proteins Bra12, SdpR, and LysRNt, respectively. The assumed or verified effects exerted by the regulators on the activities of the corresponding gene promoters are presented by vertical arrows (activation) and T-lines (repression). In the case of LysRNt, its putative role in gene transcription has been shown, as the direct impact on promoter activity was not studied (Wolański et al. 2021). The bent arrows indicate the transcription start sites (TSS) (see also Fig. S8)

In the case of SdpR, the arrangement of its binding sites in the promoter regions did not allow us to clearly predict a general function for the protein in the regulation of gene expression in the Bra-BGC. For instance, in the biosynthetically essential *bra0 - 1* region, the presence of two SdpR boxes, both upstream and downstream *bra0* and *bra1* gene TSSs, respectively, suggested a corresponding positive and negative role in gene expression (Fig. 7). However, the reporter assays revealed a negative impact of *sdpR* gene disruption on the activity of the *bra0* promoter (~ tenfold decrease in comparison to the control strain), while for *bra1*, only a slight delay in the promoter activity peak was observed (Fig. 5A). The impact exerted on the *bra0* promoter corresponds to the predicted function for the SdpR. We propose that two effects presumably exerted the observed shift in the *bra1* promoter activity, a reduction of the level of Bra12 and an absence of SdpR. The reduction of Bra12 levels might result from a pronouncedly decreased (over 100-fold) activity of the *bra12* promoter observed in the $\Delta$sdpR strain. Importantly, the positive impact of SdpR on the *bra12p* activity was also confirmed using a *sdpR* overexpression strain (Fig. S9). Further, a decreased level of Bra12 in the absence of SdpR should therefore lead to a significant reduction in *bra1* promoter activity. However, we demonstrate that a lack of SdpR leads only to a small delay in *bra1p* activity, and finally to increased levels of brasilicardins. These findings led us to suggest that SdpR may negatively, by binding to the SdpR box located downstream of the TSS of the *bra1* gene, regulate the expression of this gene promoter. In sum, the combined impact of the absence of the SdpR repressor and the decrease in Bra12 activator levels results in a slightly delayed activation of the *bra1* promoter. Importantly, using EMSA, we demonstrate that both regulators do not compete for binding to DNA fragments comprising the *bra0 - 1* intergenic region (Fig. S10). This suggests that Bra12 and SdpR may independently exert an impact on the gene expression of *bra1* (and the downstream genes *bra2*–*bra11*) probably as the result of a balance in cellular levels between both proteins. The complexity of putative regulatory functions by SdpR has been also revealed in the case of *sdpRp*. Surprisingly, despite the presence of two SdpR boxes in the *sdpRp* region, SdpR did not show any impact on its own gene promoter activity excluding the possibility of an autoregulatory function. We speculate that SdpR, which belongs to a large family of metalloregulators widespread in bacteria, may be subject to another level of regulation, related to the metal sensing as it was shown for ArsR, SmtB, and CmtT regulators of *E. coli*, *Synechococcus*, and *Mycobacterium*, respectively (Erbe et al. 1995; Xu et al. 1996; Wang et al. 2005).

Here, we demonstrate that both Bra12 and SdpR exert important, nevertheless opposite effects, on brasilicardin congener biosynthesis. Consistently, with the previous research (Schwarz et al. 2018a), we show that Bra12 positively affects the process of biosynthesis, although, in comparison to that study, we observed about a 3-times higher increase in the production titers in the host strain overproducing the Bra12 regulator (63 vs 196%, respectively) (Fig. 6). As in both studies, the *bra12* was delivered under constitutive ermE* promoter on the pIJ10257 integrative plasmid; the discrepancies in biosynthesis yields likely result from various genetic backgrounds of the host strains. Indeed, a full-length wild-type bcaAB01 fosmid (Bra-BGC plus flanking genes) was used in the previous study, while a shortened pPS1 version (only the Bra-BGC) was employed in our approach. This clearly indicates additional impact exerted by other regulatory proteins encoded in the genes flanking the Bra-BGC. Up to date, the LysRNt, and SdpR are the only, apart from Bra12, identified regulators of the brasilicardin gene cluster. Importantly, the individual overexpression of LysRNt and SdpR regulators in identical genetic backgrounds (host strain harboring pPS1) showed their negative impacts manifested by a decrease in the brasilicardin production levels (~ 27% and 64% levels of the strain harboring only the pPS1 fosmid for LysRNt and SdpR overexpression, respectively). Consistently, deletion of SdpR leads to a pronounced increase in the production levels of brasilicardin congeners (~ 397%).

In conclusion, the regulation of gene expression in the Bra-BGC seems complex and represents a still not fully understood process that requires concerted action of multiple regulators to ensure biosynthesis of the BraA compound. We assume that the intergenic *bra0 - 1* region plays a role of an essential regulatory “hot-spot” comprising binding sites for multiple regulatory proteins that control transcription of the biosynthetic genes in a coordinated way. The involvement of multiple regulator binding sites within this region may reflect the complexity of conditions of this free-living bacterium which it faces in the environment and the necessity of diversified responses to external factors to ensure tight control over gene expression in the Bra-BGC. As the putative function of the BraA during infection has not been explained so far, the range of external signals may also include the ones related to the host organism of this facultative pathogen. In addition, the feedback mechanisms including regulation of gene expression of regulatory genes by other regulators create another level of complexity and make understanding of transcriptional regulatory circuits taking place in Bra-BGC even more challenging. To contribute to this process, we propose a current model for the network of regulatory dependencies in the Bra-BGC (Fig. 7).

## Supplementary Information

Below is the link to the electronic supplementary material.

ESM 1 (PDF 4.38 MB)

ESM 2 (XLSX 71.9 KB)

ESM 3 (XLSX 84.5 KB)

ESM 4 (XLSX 4.09 MB)

## Data Availability

The gene expression data generated and analyzed in this study have been deposited in the NCBI Gene Expression Omnibus (GEO) under the accession number GSE271981. The dataset will be publicly available from 10 July 2025. Until that time, the data are available from the corresponding author upon reasonable request.

## References

[CR1] Anada M, Hanari T, Kakita K, Kurosaki Y, Katsuse K, Sunadoi Y, Jinushi Y, Takeda K, Matsunaga S, Hashimoto S (2017) Total synthesis of Brasilicardins A and C. Org Lett 19:5581–5584. 10.1021/acs.orglett.7b02728 28976203 10.1021/acs.orglett.7b02728

[CR2] Bailey TL, Boden M, Buske FA, Frith M, Grant CE, Clementi L, Ren J, Li WW, Noble WS (2009) MEME Suite: tools for motif discovery and searching. Nucleic Acids Res 37:W202–W208. 10.1093/nar/gkp335 19458158 10.1093/nar/gkp335 PMC2703892

[CR3] Bauman KD, Butler KS, Moore BS, Chekan JR (2021) Genome mining methods to discover bioactive natural products. Nat Prod Rep 38:2100. 10.1039/D1NP00032B 34734626 10.1039/d1np00032b PMC8597713

[CR4] Botas A, Eitel M, Schwarz PN, Buchmann A, Costales P, Núñez LE, Cortés J, Morís F, Krawiec M, Wolański M, Gust B, Rodriguez M, Fischer WN, Jandeleit B, Zakrzewska-Czerwińska J, Wohlleben W, Stegmann E, Koch P, Méndez C, Gross H (2021) Genetic engineering in combination with semi-synthesis leads to a new route for gram-scale production of the immunosuppressive natural product brasilicardin A. Angew Chem Int Ed 60:13536–13541. 10.1002/anie.202015852 10.1002/anie.202015852 PMC8251711 33768597

[CR5] Buchmann A, Eitel M, Koch P, Schwarz PN, Stegmann E, Wohlleben W, Wolański M, Krawiec M, Zakrzewska-Czerwińska J, Méndez C, Botas A, Núñez LE, Morís F, Cortés J, Gross H (2016) High-quality draft genome sequence of the actinobacterium *Nocardia terpenica* IFM 0406, producer of the immunosuppressant brasilicardins, using Illumina and PacBio technologies. Genome Announc 4:1391–1407. 10.1128/genomeA.01391-16 10.1128/genomeA.01391-16 PMC5159576 27979943

[CR6] Chen J, Frediansyah A, Männle D, Straetener J, Brötz-Oesterhelt H, Ziemert N, Kaysser L, Gross H (2020) New nocobactin derivatives with antimuscarinic activity, terpenibactins A-C, revealed by genome mining of *Nocardia terpenica* IFM 0406. ChemBioChem 21:2205–2213. 10.1002/cbic.202000062 32196864 10.1002/cbic.202000062 PMC7497119

[CR7] Craney A, Hohenauer T, Xu Y, Navani NK, Li Y, Nodwell J (2007) A synthetic *luxCDABE* gene cluster optimized for expression in high-GC bacteria. Nucleic Acids Res 35:e46. 10.1093/nar/gkm086 17337439 10.1093/nar/gkm086 PMC1874620

[CR8] Crooks GE, Hon G, Chandonia JM, Brenner SE (2004) WebLogo: a sequence logo generator. Genome Res 14:1188–1190. 10.1101/gr.849004 15173120 10.1101/gr.849004 PMC419797

[CR9] Ding T, Yang LJ, Zhang WD, Shen YH (2019) The secondary metabolites of rare actinomycetes: chemistry and bioactivity. RSC Adv 9:21964–21988. 10.1039/c9ra03579f 35518871 10.1039/c9ra03579f PMC9067109

[CR10] Dugar G, Herbig A, Förstner KU, Heidrich N, Reinhardt R, Nieselt K, Sharma CM (2013) High-resolution transcriptome maps reveal strain-specific regulatory features of multiple *Campylobacter jejuni* isolates. PLoS Genet 9:e1003495. 10.1371/journal.pgen.1003495 23696746 10.1371/journal.pgen.1003495 PMC3656092

[CR11] Engelbrecht A, Saad H, Gross H, Kaysser L (2021) Natural products from *Nocardia* and their role in pathogenicity. Microb Physiol 31:217–232. 10.1159/000516864 34139700 10.1159/000516864

[CR12] Erbe JL, Taylor KB, Hall LM (1995) Metalloregulation of the cyanobacterial smt locus: indentification of SmtB binding sites and direct interaction with metals. Nucleic Acids Res 23:2472–2478. 10.1093/nar/23.13.2472 7630724 10.1093/nar/23.13.2472 PMC307053

[CR13] Förstner KU, Vogel J, Sharma CM (2014) READemption-a tool for the computational analysis of deep-sequencing-based transcriptome data. Bioinformatics 30:3421–3423. 10.1093/bioinformatics/btu533 25123900 10.1093/bioinformatics/btu533

[CR14] Gasteiger E, Hoogland C, Gattiker A, Duvaud S, Wilkins MR, Appel RD, Bairoch A (2005) Protein identification and analysis tools on the ExPASy server. The Proteomics Protocols Handbook 571–607. 10.1385/1-59259-890-0:571

[CR15] Gavriilidou A, Kautsar SA, Zaburannyi N, Krug D, Müller R, Medema MH, Ziemert N (2022) Compendium of specialized metabolite biosynthetic diversity encoded in bacterial genomes. Nat Microbiol 7:726–735. 10.1038/s41564-022-01110-2 35505244 10.1038/s41564-022-01110-2

[CR16] Gross H (2007) Strategies to unravel the function of orphan biosynthesis pathways: recent examples and future prospects. Appl Microbiol Biotechnol 75:267–277. 10.1007/s00253-007-0900-5 17340107 10.1007/s00253-007-0900-5

[CR17] Hoskisson PA, Rigali S, Fowler K, Findlay KC, Buttner MJ (2006) DevA, a GntR-like transcriptional regulator required for development in *Streptomyces coelicolor*. J Bacteriol 188:5014–5023. 10.1128/JB.00307-06 16816174 10.1128/JB.00307-06 PMC1539961

[CR18] Itou H, Tanaka I (2001) The OmpR-family of proteins: insight into the tertiary structure and functions of two-component regulator proteins. J Biochem 129:343–350. 10.1093/oxfordjournals.jbchem.a002863 11226872 10.1093/oxfordjournals.jbchem.a002863

[CR19] Jakoby M, Nolden L, Meier-Wagner J, Krämer R, Burkovski A (2000) AmtR, a global repressor in the nitrogen regulation system of *Corynebacterium glutamicum*. Mol Microbiol 37:964–977. 10.1046/j.1365-2958.2000.02073.x 10972815 10.1046/j.1365-2958.2000.02073.x

[CR20] Kieser T, Bibb MJ, Chater KF, Butter MJ, Hopwood DA (2000) Practical streptomyces genetics: a laboratory manual. John Innes Foundation, Norwich, United Kingdom

[CR21] Komaki H, Tanaka Y, Yazawa K, Takagi H, Ando A, Nagata Y, Mikami Y (2000) Antitumor activity of brasilicardin A, a novel terpenoid antibiotic from *Nocardia brasiliensis*. J Antibiot 53:75–77. 10.7164/antibiotics.53.75 10.7164/antibiotics.53.75 10724013

[CR22] Komatsu K, Tsuda M, Shiro M, Tanaka Y, Mikami Y, Kobayashi J (2004) Brasilicardins B-D, new tricyclic terpernoids from actinomycete *Nocardia brasiliensis*. Bioorg Med Chem 12:5545–5551. 10.1016/j.bmc.2004.08.007 15465331 10.1016/j.bmc.2004.08.007

[CR23] Komatsu K, Tsuda M, Tanaka Y, Mikami Y, Kobayashi J (2005) SAR studies of brasilicardin A for immunosuppressive and cytotoxic activities. Bioorg Med Chem 13:1507–1513. 10.1016/j.bmc.2004.12.029 15698766 10.1016/j.bmc.2004.12.029

[CR24] Laemmli UK (1970) Cleavage of structural proteins during the assembly of the head of bacteriophage T4. Nature 227:680–685. 10.1038/227680a0 5432063 10.1038/227680a0

[CR25] Lee N, Kim W, Hwang S, Lee Y, Cho S, Palsson B, Cho BK (2020) Thirty complete *Streptomyces* genome sequences for mining novel secondary metabolite biosynthetic gene clusters. Sci Data 7:1–9. 10.1038/s41597-020-0395-9 32054853 10.1038/s41597-020-0395-9 PMC7018776

[CR26] Letunic I, Bork P (2018) 20 years of the SMART protein domain annotation resource. Nucleic Acids Res 46:D493–D496. 10.1093/nar/gkx922 29040681 10.1093/nar/gkx922 PMC5753352

[CR27] Li MZ, Elledge SJ (2007) Harnessing homologous recombination *in vitro* to generate recombinant DNA via SLIC. Nat Methods 4:251–256. 10.1038/nmeth1010 17293868 10.1038/nmeth1010

[CR28] Li YC, Chang CK, Chang CF, Cheng YH, Fang PJ, Yu T, Chen SC, Li YC, Hsiao CD, Huang TH (2014) Structural dynamics of the two-component response regulator RstA in recognition of promoter DNA element. Nucleic Acids Res 42:8777. 10.1093/NAR/GKU572 24990372 10.1093/nar/gku572 PMC4117788

[CR29] Loh KD, Gyaneshwar P, Papadimitriou EM, Fong R, Kim KS, Parales R, Zhou Z, Inwood W, Kustu S (2006) A previously underscribed pathway for pyrimidine catabolism. Proc Natl Acad Sci U S A 103:5114–5119. 10.1073/pnas.0600521103 16540542 10.1073/pnas.0600521103 PMC1458803

[CR30] Marchler-Bauer A, Bo Y, Han L, He J, Lanczycki CJ, Lu S, Chitsaz F, Derbyshire MK, Geer RC, Gonzales NR, Gwadz M, Hurwitz DI, Lu F, Marchler GH, Song JS, Thanki N, Wang Z, Yamashita RA, Zhang D, Zheng C, Geer LY, Bryant SH (2017) CDD/SPARCLE: functional classification of proteins via subfamily domain architectures. Nucleic Acids Res 45:D200–D203. 10.1093/nar/gkw1129 27899674 10.1093/nar/gkw1129 PMC5210587

[CR31] Niman SW, Buono R, Fruman DA, Vanderwal CD (2023) Synthesis of a complex brasilicardin analogue utilizing a cobalt-catalyzed MHAT-induced radical bicyclization reaction. Org Lett 25:3451–3455. 10.1021/acs.orglett.3c01019 37141632 10.1021/acs.orglett.3c01019 PMC10204089

[CR32] Novakova R, Kutas P, Feckova L, Kormanec J (2010) The role of the TetR-family transcriptional regulator Aur1R in negative regulation of the auricin gene cluster in *Streptomyces aureofaciens* CCM 3239. Microbiology (n Y) 156:2374–2383. 10.1099/mic.0.037895-0 10.1099/mic.0.037895-0 20466770

[CR33] Oren A, Garrity GM (2021) Valid publication of the names of forty-two phyla of prokaryotes. Int J Syst Evol Microbiol 71. 10.1099/ijsem.0.005056 10.1099/ijsem.0.005056 34694987

[CR34] Płachetka M, Krawiec M, Zakrzewska-Czerwińska J, Wolański M (2021) AdpA positively regulates morphological differentiation and chloramphenicol biosynthesis in *Streptomyces venezuelae*. Microbiol Spectr 9. 10.1128/spectrum.01981-21 10.1128/Spectrum.01981-21 PMC8653842 34878326

[CR35] Ramos JL, Martínez-Bueno M, Molina-Henares AJ, Terán W, Watanabe K, Zhang X, Gallegos MT, Brennan R, Tobes R (2005) The TetR family of transcriptional repressors. Microbiol Mol Biol Rev 69:326–356. 10.1128/MMBR.69.2.326-356.2005 15944459 10.1128/MMBR.69.2.326-356.2005 PMC1197418

[CR36] Rutledge PJ, Challis GL (2015) Discovery of microbial natural products by activation of silent biosynthetic gene clusters. Nat Rev Microbiol 13:509–523. 10.1038/nrmicro3496 26119570 10.1038/nrmicro3496

[CR37] Sambrook J, Russell DW (2001) Molecular cloning, a laboratory manual, the, 3rd edn. Cold Spring Horbor laboratory Press, Cold Spring Harbor Laboratory Press, Cold Spring Harbor, NY

[CR38] Santamarta I, Rodríguez-García A, Pérez-Redondo R, Martín JF, Liras P (2002) CcaR is an autoregulatory protein that binds to the *ccaR* and *cefD-cmcI* promoters of the cephamycin C-clavulanic acid cluster in *Streptomyces clavuligerus*. J Bacteriol 184:3106–3113. 10.1128/JB.184.11.3106-3113.2002 12003953 10.1128/JB.184.11.3106-3113.2002 PMC135043

[CR39] Schultz J, Milpetz F, Bork P, Ponting CP (1998) SMART, a simple modular architecture research tool: identification of signaling domains. Proc Natl Acad Sci U S A 95:5857–5864. 10.1073/pnas.95.11.5857 9600884 10.1073/pnas.95.11.5857 PMC34487

[CR40] Schwarz PN, Buchmann A, Roller L, Kulik A, Gross H, Wohlleben W, Stegmann E (2018a) The immunosuppressant brasilicardin: determination of the biosynthetic gene cluster in the heterologous host *Amycolatopsis japonicum*. Biotechnol J 13:1–12. 10.1002/biot.201700527 10.1002/biot.201700527 29045029

[CR41] Schwarz PN, Roller L, Kulik A, Wohlleben W, Stegmann E (2018b) Engineering metabolic pathways in *Amycolatopsis japonicum* for the optimization of the precursor supply for heterologous brasilicardin congeners production. Synth Syst Biotechnol 3:56–63. 10.1016/j.synbio.2017.12.005 29911199 10.1016/j.synbio.2017.12.005 PMC5884276

[CR42] Sharma CM, Hoffmann S, Darfeuille F, Reignier J, Findeiß S, Sittka A, Chabas S, Reiche K, Hackermüller J, Reinhardt R, Stadler PF, Vogel J (2010) The primary transcriptome of the major human pathogen *Helicobacter pylori*. Nature 464:250–255. 10.1038/nature08756 20164839 10.1038/nature08756

[CR43] Shigemori H, Komaki H, Yazawa K, Mikami Y, Nemoto A, Tanaka Y, Sasaki T, In Y, Ishida T, Kobayashi JNI (1998) Brasilicardin A. A novel tricyclic metabolite with potent immunosuppressive activity from actinomycete *Nocardia brasiliensis*. J Org Chem 63:6900–6904. 10.1021/jo9807114 11672311 10.1021/jo9807114

[CR44] Tanaka A, Takano Y, Ohnishi Y, Horinouchi S (2007) AfsR recruits RNA polymerase to the afsS promoter: a model for transcriptional activation by SARPs. J Mol Biol 369:322–333. 10.1016/j.jmb.2007.02.096 17434533 10.1016/j.jmb.2007.02.096

[CR45] Tsypik O, Makitrynskyy R, Yan X, Koch HG, Paululat T, Bechthold A (2021) Regulatory control of rishirilide(S) biosynthesis in Streptomyces bottropensis. Microorganisms 9:1–15. 10.3390/microorganisms9020374 10.3390/microorganisms9020374 PMC7917814 33673359

[CR46] Usui T, Nagumo Y, Watanabe A, Kubota T, Komatsu K, Kobayashi J, Osada H (2006) Brasilicardin A, a natural immunosuppressant, targets amino acid transport system L. Chem Biol 13:1153–1160. 10.1016/j.chembiol.2006.09.006 17113997 10.1016/j.chembiol.2006.09.006

[CR47] Wang Y, Hemmingsen L, Giedroc DP (2005) Structural and functional characterization of *Mycobacterium tuberculosis* CmtR, a PbII/CdII-sensing SmtB/ArsR metalloregulatory repressor. Biochemistry 44:8976–8988. 10.1021/bi050094v 15966722 10.1021/bi050094v

[CR48] Wolański M, Krawiec M, Schwarz PN, Stegmann E, Wohlleben W, Buchmann A, Gross H, Eitel M, Koch P, Botas A, Méndez C, Núñez LE, Morís F, Cortés J, Zakrzewska-Czerwińska J (2021) A novel LysR-type regulator negatively affects biosynthesis of the immunosuppressant brasilicardin. Eng Life Sci 21:4–18. 10.1002/elsc.202000038 33531886 10.1002/elsc.202000038 PMC7837296

[CR49] Wolański M, Łebkowski T, Kois-Ostrowska A, Zettler J, Apel AK, Jakimowicz D, Zakrzewska-Czerwińska J (2016) Two transcription factors, CabA and CabR, are independently involved in multilevel regulation of the biosynthetic gene cluster encoding the novel aminocoumarin, cacibiocin. Appl Microbiol Biotechnol 100:3147–3164. 10.1007/s00253-015-7196-7 26637421 10.1007/s00253-015-7196-7

[CR50] Xu C, Shi W, Rosen BP (1996) The chromosomal *arsR* gene of *Escherichia coli* encodes a trans-acting metalloregulatory protein. J Biol Chem 271:2427–2432. 10.1074/jbc.271.5.2427 8576202 10.1074/jbc.271.5.2427

[CR51] Yoshimura F, Itoh R, Torizuka M, Mori G, Tanino K (2018) Asymmetric total synthesis of brasilicardins. Angew Chem Int Ed 57:17161–17167. 10.1002/anie.201811403 10.1002/anie.201811403 30383323

