# Supplementary material for: Multiple regulators control the biosynthesis of brasilicardin in Nocardia terpenica
Source: Appl Microbiol Biotechnol. 2025 Jun 24;109(1):150. doi: 10.1007/s00253-025-13485-3 (PMC12185567; doi:10.1007/s00253-025-13485-3)
Supplement: Supplementary file 1 — (PDF 4.38 MB) [file 253_2025_13485_MOESM1_ESM.pdf]

Applied Microbiology and Biotechnology

Multiple regulators control the biosynthesis of brasilicardin in *Nocardia terpenica*

Running title: Multilevel regulation of gene expression of brasilicardin A biosynthetic gene cluster

Marcin Wolański[1]*, Michał Krawiec[1], Kay Nieselt[2], Tobias Schwarz[2], Dilek Dere[2], Bernhard Krismer[3],

Carolina Cano-Prieto[4], Harald Gross[4] and Jolanta Zakrzewska-Czerwińska[1] * Corresponding author,

marcin.wolanski@uwr.edu.pl, tel. +48 71 375 62 43

(1) Faculty of Biotechnology, University of Wrocław, Wrocław, Poland.

(2) Institute for Bioinformatics and Medical Informatics, University of Tübingen, Tübingen, Germany.

(3) Interfaculty Institute for Microbiology and Infection Medicine Tübingen, Infection Biology Unit, University of Tübingen, Tübingen, Germany.

(4) Pharmaceutical Institute, Department of Pharmaceutical Biology, University of Tübingen, Tübingen, Germany

The list of supplementary materials:

1. Supplementary figures

Fig. S1. Purification of recombinant proteins.
Fig. S2. Preliminary identification of the KstR protein binding sites within the bcaAB01 fosmid.
Fig. S3. Preliminary identification of the SdpR protein binding sites within the bcaAB01 fosmid.
Fig. S4. Preliminary identification of the Bra12 protein binding sites within the bcaAB01 fosmid.
Fig. S5. Preliminary identification of the OmpR protein binding sites within the bcaAB01 fosmid.
Fig. S6. Supplementary identification of regulatory protein target promoters within the Bra-BGC.
Fig. S7. Detailed analysis of SdpR binding within promoter regions.
Fig. S8. Transcription Start Sites (TSSs) in selected promoter regions within the Bra-BGC.
Fig. S9. Impact of the SdpR regulator on promoter activities.
Fig. S10. Simultaneous binding of Bra12 and SdpR to the bra0-1 intergenic region.
Fig. S11. Expression tracks for the Bra-BGC.
Fig. S12. Expression of *bra12* and *sdpR* regulatory gene expression in *A. japonicum* producer strains.

2. Supplementary tables

Table S1. Plasmids and strains (see below in this file).
Table S2. Oligonucleotides (see below in this file).
Table S3A-B. RNA-seq gene expression. **(A)** Raw (Raw counts) and **(B)** normalized (RPKM) expression data (see in SI Excel file).
Table S4. In silico analysis of predicted transcriptional regulators of Bra-BGC (see below in this file).
Table S5A-E. Homologs of regulatory proteins in *Nocardia terpenica* IFM0406. **(A)** Homologs of KstR; **(B)** Homologs of SdpR; **(C)** Homologs of Bra12; **(D)** Homologs of LysRNt; **(E)** Homologs of OmpR (see in SI Excel file).
Table S6. Gene transcriptional start site (see in SI Excel file).
Table S7. Bioinformatic tools (see below in this file).

3. Supplementary Materials and Methods

DNA constructs and strains
Protein expression and purification
Protein-DNA interactions
Internet links to sequence resources

4. Supplementary References

See in the bottom of this file.

Supplementary figures

Fig. S1. Purification of recombinant proteins.

(A) Schematic depiction of protein domain organizations based on SMART and conserved domain (CD) searches (see Tab. S4). The SMART and Protein Family (PF) numbers of the identified protein domains are given. The DNA-binding domain has been indicated with blue and other domains according to the legend. **(B)** SDS-PAGE analysis of purified recombinant proteins. The His-tagged proteins were purified using metal affinity resins, HiTrap Talon® crude column (1 ml), or His-Select® Nickel Affinity Gel, as described in detail in the SI. The elution fractions were collected while the resins were washed with an increasing gradient of buffer B (2-50%) containing 500 mM imidazole. The arrows indicate bands representing the corresponding recombinant proteins together with their molecular weights. M: protein weight marker (#26610, Thermo Fisher Scientific).

Fig. S2. Preliminary identification of the KstR protein binding sites within the bcaAB01 fosmid.

EMSA. The fosmid was digested independently with four restriction enzymes (Acc65I, BamHI, EcoRI, and XhoI) and incubated in the presence of 100 and 1000 nM concentrations of KstR-His protein, followed by electrophoresis on an agarose gel. The DNA was visualized by soaking the gel in an ethidium bromide solution. The red and yellow rectangles indicate vanished and shifted bands, respectively. M: DNA molecular weight marker (#SM0323, Thermo Fisher Scientific).

The bottom panel represents a graphical representation of the brasilicardin biosynthetic gene cluster on the bcaAB01 fosmid and identified vanished and shifted DNA fragments (red and yellow bars, respectively). The numbers next to those bars show the nucleotide positions on bcaAB01. Numbers above the genes are NCBI accession numbers in "AWN90_RS…" format. The gene promoters selected for further analysis are indicated by a black vertical arrow.

Fig. S3. Preliminary identification of the SdpR protein binding sites within the bcaAB01 fosmid.

Electrophoretic mobility shift assay. The figure represents two experiments. In each of those, the fosmid was independently digested with a set of different restriction enzymes and incubated in the presence of increasing concentrations of SdpR-His protein (10 and 100 nM – experiment 1; 100 and 1000 nM – experiment 2). Incubation was followed by electrophoresis on an agarose gel, and DNA visualization was conducted by soaking the gel in ethidium bromide solution. The red and yellow rectangles on the gels indicate vanished and shifted bands, respectively. M1, M2, and M – DNA molecular weight markers (λ/PstI, # 3530-500, A&A Biotechnology; #SM0323, Thermo Fisher Scientific – experiment 1, and #SM0311, Thermo Fisher Scientific – experiment 2, respectively).

The bottom panel represents a graphical analysis of the results. The numbers next to those bars show the nucleotide positions on bcaAB01. Numbers above the genes are NCBI accession numbers in "AWN90_RS…" format. The gene promoters selected for further analysis are indicated by a black vertical arrow.

Fig. S4. Preliminary identification of the Bra12 protein binding sites within the bcaAB01 fosmid.

Electrophoretic mobility shift assay. The fosmid was independently digested with four restriction enzymes (Acc65I, BamHI, EcoRI, and XhoI) and incubated in the presence of 50 and 500 nM concentrations of Bra12-His protein, followed by electrophoresis in an agarose gel. The DNA was visualized by soaking the gel in an ethidium bromide solution. The red and yellow rectangles indicate vanished and shifted bands, respectively. M1, M2 – DNA molecular weight markers (#SM0311, Thermo Fisher Scientific; #SM1193, Thermo Fisher Scientific).

The bottom panel represents a graphical analysis of the results. The numbers next to those bars show the nucleotide positions on bcaAB01. Numbers above the genes are NCBI accession numbers in "AWN90_RS…" format. The gene promoters selected for further analysis are indicated by a black vertical arrow.

Fig. S5. Preliminary identification of the OmpR protein binding sites within the bcaAB01 fosmid.

Electrophoretic mobility shift assay. The fosmid was independently digested with four restriction enzymes (Acc65I, BamHI, EcoRI, and XhoI) and incubated in the presence of 100 and 750 nM concentrations of OmpR-His protein, followed by electrophoresis on an agarose gel. The DNA was visualized by soaking the gel in an ethidium bromide solution. The red and yellow rectangles indicate vanished and shifted bands, respectively.

M1, M2: DNA molecular weight markers (λ/PstI, # 3530-500, A&A Biotechnology; #SM0311, Thermo Fisher Scientific; #SM1193, Thermo Fisher Scientific).
The bottom panel represents a graphical analysis of the results. Numbers above the genes are NCBI accession numbers in "AWN90_RS…" format.

Fig. S6. Supplementary identification of regulatory protein target promoters within the Bra-BGC.
(A) Electrophoretic mobility shift assays (EMSA). Recombinant proteins (KstR-His and SdpR-His) were incubated with preselected promoter regions (see Fig. S2-5) amplified by PCR. In the assays, constant amounts of unlabeled DNA and varying concentrations of proteins were used, as indicated. To confirm specific binding and compare binding to different DNA fragments, the reaction mixtures were spiked additionally with the DNA comprising the *33140* gene promoter region (negative control, NC) (KstR and SdpR panels) and other DNA fragments comprising promoters of the Bra-BGC (only SdpR panel). Vertical black bars indicate protein-DNA complexes and black and gray arrows indicate unbound DNA fragments. The DNA fragments and corresponding protein-DNA complexes are color-coded. **(B)** Graphical depiction of DNA fragments used in shift assays. The corresponding primers are listed in Tab. S2.

Fig. S7. Detailed analysis of SdpR binding within promoter regions.
Electrophoretic mobility shift assay (EMSA). The identification of SdpR binding sites within the *sdpR*, *bra0-1,* and *bra12* promoter regions was shown in panels **(A)**, **(B),** and **(C)**, respectively. The recombinant SdpR-His protein was incubated with the subsets of PCR-amplified DNA fragments (primer list in Tab. S2). In the assays, constant amounts of unlabeled DNA and varying concentrations of the protein were used, as indicated. The *33140* gene promoter region (33410p) and sdpRp_4-1 fragments served as negative controls. Vertical black bars indicate protein-DNA complexes and black and gray arrows indicate unbound DNA fragments. All panels contain a graphical depiction of the results. In these drawings, the green '+' symbols indicate the interactions of SdpR-His with the corresponding DNA fragments, and the red '–' symbols represent the opposite observations, respectively. The regions used for *in silico* identification of binding sequences are marked with orange.

Fig. S8. Transcription Start Sites (TSSs) in selected promoter regions within the Bra-BGC.
RNA-seq. The TSSs are marked with bald lowercase letters and bent arrows. The first or the last twenty nucleotides of the gene sequences surrounding promoter regions are underlined; stop and start codons of corresponding genes are highlighted in red and green color, respectively. For original data, see Table S6.

Fig. S9. Impact of the SdpR regulator on promoter activities.
Luciferase assays. To study the impact of SdpR overexpression, the measurements were conducted using *S. coelicolor* M1154 heterologous strains harboring *bra12p* and *sdpRp* gene promoters delivered onto pFLUX integrative plasmids, and replicating the pUWL201 plasmid overexpressing SdpR (+sdpR); the strains containing an empty pUWL201 plasmid served as the controls (pUWL). The impact of *sdpR* gene deletion was shown using a strain carrying the pFLUX plasmid with *sdpRp,* and the bcaAB01_ΔsdpR fosmid (bottom panel). The luminescence readings were normalized against the OD_{600} of the corresponding cultures grown on a solid DNA medium for 11 days.

Fig. S10. Simultaneous binding of Bra12 and SdpR to the bra0-1 intergenic region.
(A) Electrophoretic mobility shift assays (EMSA). (left) The recombinant proteins (Bra12-His and SdpR-His) were incubated with the *bra0-1* intergenic region (^{32}P-radiolabeled bra0-1p_5-6 fragment). A constant amount of DNA (~ 5 mol) and the concentration of Bra12-His protein (blue bar) were used, and the SdpR-His protein was added at increasing concentrations (orange triangles), as indicated. Black vertical bars indicate protein-DNA complexes; a black arrow indicates unbound DNA. (right) Graphical summary of the EMSA and a model depicting simultaneous binding of Bra12 and SdpR to *bra0-1* intergenic region. The proteins are represented by colored circles (blue: Bra12, orange: SdpR), and the primers and the TSSs are shown with plain arrows and bent arrows, respectively. **(B)** competition EMSA. (left) The Bra12-His and SdpR-His at constant concentrations were incubated with the constant amount of the ^{32}P-labeled DNA fragment (bra0-1p_13-6) comprising the *bra0-1* intergenic region. The "cold-target DNA" competitors (ctDNA) were added to the reaction mixtures at 25 nM final concentration. Black vertical bars indicate protein-DNA complexes (marked also with dotted lines); a black arrow indicates unbound DNA. (right) Graphical summary of the assay.

Radiolabeled DNA is indicated with asterisks (*). The green '+' and the red '–' symbols indicate the ability and lack of ability, respectively, of the corresponding 'cold-target DNA' fragment to outperform ^{32}P-labeled DNA. Primers are indicated by short arrows. The numbers in brackets represent fragment sizes (bp).

Fig. S11. Expression tracks for the Bra-BGC.

Raw gene expression data for *N. terpenica* IFM0406 grown for 33 and 48 h. Two replicates, R1 and R2, are shown. The expression tracks shown in blue and pink refer to normal RNA-seq and +TEX 5'-enriched libraries. The unit of the Y axis is coverage. The black and gray arrows on the left side show sense and anti-sense DNA strands of the *N. terpenica* chromosome. Please note that due to software limitations, gene orientations are shown in a "natural" manner and appear on the chromosome sequence, and not the way we used throughout the article.

Fig. S12. Expression of *bra12* and *sdpR* regulatory gene expression in *A. japonicum* producer strains.

Transcriptional analysis of *bra12* and *sdpR* was conducted on samples collected at the 72-hour time point from two or three independent cultures of *A. japonicum* grown in liquid medium. Relative gene expression (RQ) levels were determined by RT-qPCR, using the sigma factor homolog *sigB* as a reference gene and the *A. japonicum::bcaAB01* chromosome (RQ = 1) as a calibrator. The graphs in panels **(A)** and **(B)** display the Y-axis in linear and logarithmic scales, respectively.

Supplementary tables

Table S1. Plasmids and strains.

Amp^R – ampicillin resistance, Apr^R – apramycin resistance, Hyg^R – hygromycin resistance, Kan^R – kanamycin resistance, Cam^R – chloramphenicol resistance, Tet^R – tetracycline resistance, Tsr^R – thiostrepton resistance

**Plasmids (and fosmids)**		
Construct name {abbreviation used in the text}	Description	Source / Reference
pET-21a(+)	Expression vector for the production of recombinant proteins with C-terminally fused 6xHis tag; Amp^R	Laboratory stock / (Merck)
pET-21a(+)kstR	pET-21a(+) derivative for expression of KstR-His recombinant protein; Amp^R	This study
pET-21a(+)sdpR	pET-21a(+) derivative for expression of SdpR-His recombinant protein; Amp^R	This study
pET-21a(+)bra12	pET-21a(+) derivative for expression of Bra12-His recombinant protein; Amp^R	This study
pET-21a(+)ompR	pET-21a(+) derivative for expression of OmpR-His recombinant protein; Amp^R	This study
pFLUX	Reporter integrating vector containing promoterless luciferase operon *luxCDAEB;* Apr^R	(Craney et al. 2007)
pFLUX-bra0p	pFLUX derivative containing *bra0* gene promoter region in front of *luxCDAEB* gene operon; Apr^R	This study
pFLUX-bra1p	pFLUX derivative containing *bra1* gene promoter region in front of *luxCDAEB* gene operon; Apr^R	This study
pFLUX-bra12p	pFLUX derivative containing *bra12* gene promoter region in front of *luxCDAEB* gene operon; Apr^R	This study
pFLUX-sdpRp	pFLUX derivative containing *sdpR* gene promoter region in front of *luxCDAEB* gene operon; Apr^R	This study
pUWL201	Replicative *Streptomyces* expression vector based on the strong, constitutive ermE promoter used for expression; Tsr^R, Amp^R	Laboratory stock / (Doumith et al. 2000)
pUWL201HX	pUWL201 derivative containing Tsr^R, Amp^R and Hyg^R resistance cassettes	This study
pUWL201HX_SdpR_MK	pUWL201HX derivative containing *sdpR* gene cloned downstream p*ermE** derived from pIJ10257; Tsr^R, Amp^R, Hyg^R	This study
pIJ10257 {pIJ}	Integrative *Streptomyces* expression vector based on the strong, constitutive *ermE* promoter (p*ermE*); Hyg^R	Laboratory stock / (Hong et al. 2005)
pPSbra12 {pIJ_bra12}	Integrative vector pIJ10257 derivative carrying *bra12* gene under the control of p*ermE**; Hyg^R	(Schwarz et al. 2018)
pIJ10257_sdpR {pIJ_sdpR}	Integrative vector pIJ10257 derivative carrying *sdpR* gene under the control of p*ermE**; Hyg^R	This study
bcaAB01	pCC1FOS (Epicentre), initial fosmid containing the brasilicardin biosynthetic gene cluster with the flanking regions; Cam^R resistance exchanged with Kan^R.	(Schwarz et al. 2018)
pPS1	Truncated bcaAB01 derivative, carrying the brasilicardin biosynthetic gene cluster (*bra0-bra11*) and the bra12 gene; Kan^R	(Schwarz et al. 2018)
bcaAB01_ΔsdpR	bcaAB01 derivative, harboring scar deletion of the *sdpR* gene; Kan^R	This study
bcaAB01_Δbra12	bcaAB01 derivative, harboring scar deletion of the *bra12* gene; Kan^R	This study
**Strains**		
Name	Description	Source / Reference
E. coli DH5α	Φ80Δ*lacZM15 recA1 endA1 gyrAB thi-1 hsdR17*(rK-mK+) *supE44 relA1 deoR* Δ(*lacZYA-argF*)*U169 phoA*	Laboratory stock / (Promega)

Escherichia coli ET12567/pUZ8002	*E. coli: dam*, *dcm*, *hsdS*, Cam^R, Tet^R containing plasmid pUZ8002: *tra*, Kan^R, *RP4 23*	Laboratory stock / (MacNeil et al. 1992; Paget et al. 1999)
E. coli Rosetta™ 2(DE3)	F^- *ompT hsdS*$_B$(r_B^- m_B^-) *gal dcm* (DE3) pRARE2 (Cam^R)	Laboratory stock / (Merck)
E. coli BW25113/pIJ790	Δ(*araD-araB*)*567* Δ*lacZ4787*(*::rrnB4*) *lacIp-40000*(*lacI*^{q}) λ^- *rpoS369*(Am) *rph 1* Δ(*rhaD rhaB*)*568 hsdR514* on the bacterial chromosome; *oriR101 repA1001*(Ts) *araBp-gam-be-exo* on the pIJ790 plasmid	Laboratory stock / (Gust et al. 2003)
Amycolatopsis japonicum MG417-CF17	Wild type	(Nishikiori et al. 1984)
A. japonicum::bcaAB01	Wild type *A. japonicum* with chromosomally integrated bcaAB01	(Schwarz et al. 2018)
A. japonicum::bcaAB01_Δbra12	Wild type *A. japonicum* with chromosomally integrated bcaAB01_Δbra12	This study
A. japonicum::bcaAB01_ΔsdpR	Wild type *A. japonicum* with chromosomally integrated bcaAB01_ΔsdpR	This study
A. japonicum::pPS1+pIJ	Wild type *A. japonicum* with chromosomally integrated pPS1 fosmid and pIJ10257 empty vector	(Wolański et al. 2021)
A. japonicum::pPS1+pIJ_bra12	Wild type *A. japonicum* with chromosomally integrated pPS1 fosmid and pIJ10257_bra12 vector	This study
A. japonicum::pPS1+pIJ_sdpR	Wild type *A. japonicum* with chromosomally integrated pPS1 fosmid and pIJ10257_sdpR vector	This study
S. coelicolor M1154	ΔSCP1, ΔSCP2, Δact, Δred, Δcpk, Δcda, rpoB[C1298T] rpsL[A262G]	Laboratory stock / (Gomez-Escribano and Bibb 2011)
M1154:: bcaAB01+bra0p-lux	M1154 with bcaAB01 and pFLUX-bra0p integrated into C31 and BT1 sites, respectively	This study
M1154:: bcaAB01_Δbra12+bra0p-lux	M1154 with bcaAB01_Δbra12 and pFLUX-bra0p integrated into C31 and BT1 sites, respectively	This study
M1154:: bcaAB01_ΔsdpR+bra0p-lux	M1154 with bcaAB01_ΔsdpR and pFLUX-bra0p integrated into C31 and BT1 sites, respectively	This study
M1154:: bcaAB01+bra1p-lux	M1154 with bcaAB01 and pFLUX-bra1p integrated into C31 and BT1 sites, respectively	This study
M1154:: bcaAB01_Δbra12+bra1p-lux	M1154 with bcaAB01_Δbra12 and pFLUX-bra1p integrated into C31 and BT1 sites, respectively	This study
M1154:: bcaAB01_ΔsdpR+bra1p-lux	M1154 with bcaAB01_ΔsdpR and pFLUX-bra1p integrated into C31 and BT1 sites, respectively	This study
M1154:: bcaAB01+bra12p-lux	M1154 with bcaAB01 and pFLUX-bra12p integrated into C31 and BT1 sites, respectively	This study
M1154:: bcaAB01_Δbra12+bra12p-lux	M1154 with bcaAB01_Δbra12 and pFLUX-bra12p integrated into C31 and BT1 sites, respectively	This study
M1154:: bcaAB01_ΔsdpR+bra12p-lux	M1154 with bcaAB01_ΔsdpR and pFLUX-bra12p integrated into C31 and BT1 sites, respectively	This study
M1154:: pUWL+bra12p-lux	M1154 with self-replicating empty pUWL201HX plasmid and pFLUX-bra12p integrated into BT1 site	This study
M1154:: pUWLsdpR +bra12p-lux	M1154 with self-replicating pUWL201HXsdpR plasmid and pFLUX-bra12p integrated into BT1 site	This study
M1154:: pUWL+sdpRp-lux	M1154 with self-replicating empty pUWL201HX plasmid and pFLUX-sdpRp integrated into BT1 site	This study
M1154:: pUWLsdpR +sdpRp-lux	M1154 with self-replicating pUWL201HXsdpR plasmid and pFLUX-sdpRp integrated into BT1 site	This study

Table S2. Oligonucleotides

Name	Sequence (5’>3’)	Application
**Cloning**		
KstR pET fwd	CTGCTCCATATGATGAGTGAGCGGCGGGAAGCG	Cloning of *kstR* gene into pET-21a(+) vector
KstR pET rev	CTGCTGCTCGAGATGAGTGGGGGCGTGCC	
SdpR pET fwd	CGTGCTCATATGGTGACAGTAGCGTTCGACGTTCTC	Cloning of *sdpR* gene into pET-21a(+) vector
SdpR pET rev	CTGCTGCTCGAGTGACGTTTCTCCTCGTTCGGGA	
AfsRforpET	CATATGTCACTGGTTCGGCTGGG	Cloning of *bra12* gene into pET-21a(+) vector
AfsRrevpET	GTCGACTCCGGAACACTGCGTGCACG	
mprA3_NdeI	GCGCACCATATGCGTTTATTGATCGTGGAGGACG	Cloning of *ompR* gene into pET-21a(+) vector
mprA3_XhoI	CGCGCGCTCGAGCATGTATTCCAGCCGGTAGC	
SdpR pET fwd	CGTGCTCATATGGTGACAGTAGCGTTCGACGTTCTC	Cloning of *sdpR* gene into pIJ10257 vector
sdpR_pIJ_rev	TACTACCTCGAGTCATGACGTTTCTCCTCGTTCGGG	
bla_P1	AATCTAAAGTATATATGAGTAAACTTGGTCTGACAGTTA TGTAGGCTGGAGCTGCTTC	Cloning of Hyg^R casstte into pUWL201
bla_P2	CCCTGATAAATGCTTCAATAATATTGAAAAAGGAAGAG TATTCCGGGGATCCGTCGACC	
FP_ermEsdpR	GCCGGCACTTCGTGCAGGCGGGTACCAGCCCGACCC	Cloning of *ermE**p into pUWL201HX vector
RP_ermEsdpR	ACGCTACTGTCATATGGGGCCTCCTGTTCTAGAC	
FP_sdpRMK	GCCCCATATGACAGTAGCGTTCGACGTTCTC	Cloning of *sdpR* gene into pUWL201HX vector
RP_sdpRMK	AGTGGATCCCCCGGGCTGCATCAGTGGTGGTGGTGGT GGTGTGACGTTTCTCCTCGTTCGGGATT	
doxgn500_fwd	TTGATATCCAGCAGACCAGCAGCAGA	Cloning *bra0p* into pFLUX. vector
doxgn500_rev	TTGGTACCCGATGACCCCTCCTGGC	
bca1up+500_fwd	TTGATATCGAAAGGTCATGTCGGTGTGC	Cloning *bra1p* into pFLUX. vector
bca1up+500_rev	TTGGTACCCACCGGACCCTCCGC	
AfsR500_fwd	GATATCCTGCTCGAGGGCGAACT	Cloning *bra12p* into pFLUX. vector
AfsR500short_rev	CATATGATAACCTCCCGGATCGAAAACT	
FPlux_promsdpR	GTACTTCGCGAAAGCTTGATATCCCTATCGCGCGGGCC	Cloning *sdpRp* into pFLUX. vector
RPlux_promsdpR	GAACGAGATCTTCTTCGTCATATGAACCGCCAGTGTGCC T	
del_SdpR_for	ACAGTAGCGTTCGACGTTCTCGTCGAGCCGAATCGCCG CTTAATTAAGCAAAATCCTGTATATCGTG	Deletion of *sdpR* gene on bcaAB01 fosmid
del_SdpR_rev	TCTCCTCGTTCGGGATTCGCGGGCTCCGGCGGGGCGTC GATTAATTAAGGAACTTCGGAATAGGAAC	
del afsR fwd	ATGATCCAGAAGCCTTCTGGTGCACCAGTTTTCGATCCG TTAATTAAGGAACTTCGGAATAGGAAC	Deletion of *bra12* gene on bcaAB01 fosmid
del afsR_rev	CTATCCGGAACACTGCGTGCACGGCAGGATCACCGGGC TTTAATTAAGCAAAATCCTGTATATCGTGC	
**EMSA and DNase I footprinting** (number used in the text)		
**DNA fragments used to study the interaction of KstR protein**		
kstRp_rv2	GCTTCCCGCCGCTCACTCAT	kstRp region
kstRp_fw2	TCATGCACGCCCAGCAGGACC	
kstRp1_rv	CGACGACGAAGGATCGGGCCAT	33470p region
kstR1_fw	GCAGCGCGGCGACGGCTT	
sigC-Ip_fw1	CCCGCGGCCAGGGCAAG	33410p region
sigC-Ip_rv1	CCCTATGGTCGCGCAGACCCG	
bca1-0p_fw1	GGTCGCAGGCATCGATGACCCC	bra0-1p region
bca1-0p_rv1	CCGCCGCCCTCGCCAC	
**sdpRp region subfragments**		
SdpR_check_for (1)	GCCGCCGAGGTCTGCGGCT	
sdpRp_2rev_foot (2)	GGACAACCCGAGCCGAGTGGCCA	As above

sdpRp_3fw (3)	ACGCGACGGCGGCG	As above
SigC_rev_SpeI (4)	ACTAGTTCAGCCCACGCCGCC	As above
sdpRp_2fw (5)	GCCTTGACGGGAATATAACC	As above
sdpRp_1rv (6)	CGTCGAACGCTACTGTCACAACCGC	As above
FP_prom_sdpR (7)	AACCGCCAGTGTGC	As above
sdpR_fw1 (8)	GTCGACTCCATCCGCTCCGCG	As above
sdpRp_1rv (9)	CGTCGAACGCTACTGTCACAACCGC	As above
**bra0-1p region subfragments**		
bca1-0p_fw1 (1)	GGTCGCAGGCATCGATGACCCC	As above
TSS_bca0_2 (2)	TGACCGGTTTCGATGTTATCCAGA	As above
bca1-0_fw_Kpn2 (3)	CCGGACAATAATTTCTGGATAAC	As above
SdpRbox1MisA_up(4)	GTTTATCCTAAATTTATCCGC	As above
short500bca1SLIC fwd (5)	GTACTTCGCGAAAGCTTGATATCTGATATTCGAGCAGC AGCTCG	As above
bca1-0p_rv2 (6)	CCGACCTCCACCCCGTACCG	As above
bca1_fwd (7)	CGGTCATATTGCCCGTGATCG	As above
RT_Rev_bca1 (8)	CGTCATGTCCGGATAGTCGTT	As above
bca1-0p_fw3 (9)	ACGGGGTGGAGGTCGGAC	As above
bca1-0p_rv1 (10)	CCGCCGCCCTCGCCAC	As above
RT_For_bca1 (11)	TGGTGAATTCCGGAGAATGG	As above
bca1up500NdeI_rev (12)	CATATGCACCGGACCCTCCGC	As above
bca1-0_fw_NcoI (13)	CATGGTATGTCGCGGATCCG	As above
TSS_bca0_1 (14)	CCGCTCGTTGGGCTG	As above
SdpRbox1MisA_dwn (15)	GCGGATAAATTTAGGATAAAC	As above
bca1-0_fw2 (16)	ATCCCGGCATACATTCGGTACGG	As above
**bra12p region subfragments**		
afsRp_rv3 foot (1)	GGCCGTCCAGCCGCACG	As above
afsRp_rv1 (2)	AGCCCCAGCCGAACCAGTGACAT	As above
TSS afsR inner (3)	CCTGCTGCTTGGGCTGAT	As above
TSS afsR 3 (4)	CTGCCGCTGCCGTGA	As above
TSS afsR 1 (5)	CTTCTGGTGCACCAGTTTTCG	As above
afsRp_rv_SmaI (6)	GGGTGTGGATAGTCAGTATGTC	As above
TSS afsR 2 (7)	GACATACTGACTATCCACACCCG	As above
afsRp_fw1 (8)	GGTCGCCGCCGGGTACG	As above
afsRp_fw2 (9)	GCGCGACGCCATCGACGG	As above
afsRp_rv2 (10)	CAGCGGCAGGCCGTCGATG	As above
AfsR500rev (11)	GGCCCGGTAGACCTCGAGG	As above
AfsR500for (12)	TCTAGAGACAGCCGCCTGGGCC	As above
AfsRboxMeme3_up (13)	GCCGCTGCCGTGACCGGAT	As above
AfsRboxMeme1_dw (14)	CGGCATAAATTTTGGGTA	As above
afsRp_up_M1M3 (15)	CCGGATTATCAGGGTATTACC	As above
AfsRboxMem1_up (16)	TACCCAAAATTTATGCCG	As above
**qPCR**		
RT_SdpR_fwd	GCCGCATTCTGGATTTGCT	qPCR analysis, *sdpR* gene
RT_SdpR_rev	CACGCGCAGATGTTTCGAG	
RT_AfsR_fwd	CCATCGATCAGGGCAATCAC	qPCR analysis, *bra12* gene
RT_AfsR_rev	GCCGTCATCAACAGGATTCG	
qRT-sigB fwd	ACCAGATCGGCAAGCACTTC	qPCR analysis, *sigB* (sigma factor) gene
qRT-sigB rev	CTGACGCAGCTTCGACATGA	

Table S3A-B. (see in SI Excel file).

Table S4. In silico analysis of predicted transcriptional regulators of Bra-BGC

Protein	Conserved domains*	MW** pI	Occurrence ***	Paralogs in *N. terpenica* ****	NCBI accession no. AWN90_RS (uniprot no.)
**KstR**	TetR/AcrR family regulator; N-terminal DBD (TetR_N, PF00440); C-terminal RD (TetR_C_6, PF13977)	22.2 kDa 6.53	Actinomycetota (*Streptomycetales, Corynebacteriales*) and Proteobacteria (γ-proteobacteria) phyla; many in Archea.	1	33480 (A0A164MP60)
additional information on KstR	helix-turn-helix (HTH) confers a DNA-binding function				
**SdpR**	SdpR/ArsR family metalloregulator; N-terminal through central DBD (HTH_ARSR, SM000418)	12.8 kDa 6.15	Actinomycetota (*Streptomycetales*, *Corynebacteriales*, *Pseudonocardiales*, *Micromonosporaceae*, *Streptosporangiales*, Micrococcales), Proteobacteria (α-proteobacteria), Firmicutes (*Bacillales*) phyla; few in Archea	2	33420
additional information on SdpR	winged-helix-turn-helix (wHTH) confers a DNA-binding function				
**Bra12**	AfsR/SARP family regulator; N-terminal DBD (Trans_reg_C, PF00486); central activator domain (BTAD, SM001043); C-terminal RD, ADP binding domain (NB-ARC, PF00931)	66.1 kDa 8.16	Actinomycetota (*Streptomycetales*, *Streptosporangiales*, *Pseudonocardiales*) phylum	21	33345 (A0A164MN84)
additional information on Bra12	winged-helix-turn-helix (wHTH) confers a DNA-binding function				
**LysRNt**	LysR-type family regulator; N-terminal DBD (HTH_1, PF00126); C-terminal RD, Co-inducer binding domain (LysR_substrate, PF03466)	31.8 kDa 5.54	Actinomycetota (*Streptomycetales*, *Micrococcales*, *Pseudonocardiales*, *Streptosporangiales*), Proteobacteria (α-proteobacteria) Phyla	14	33340

**OmpR**	OmpR superfamily regulator; N-terminal RD, receiver domain (REC, SM000448), C-terminal putative DBD (Trans_reg_C, SM000862)	25.1 kDa 5.90	Actinomycetota (*Streptomycetales*, *Micrococcales*, *Corynebacteriales*, *Pseudonocardiales*), Firmicutes phyla	27	33330 (A0A164MN41)
additional information on OmpR	winged-helix-turn-helix (wHTH) confers a DNA-binding function				

DBD – DNA-binding domain, RD – regulatory domain

* The conserved domain families were identified using SMART and CD-search tools (see M&M). Predicted functions are designated with DBD and RD for the DNA-binding, and regulatory domain, respectively. The corresponding specific domains are given in brackets.

** Calculated using ProtParam.

*** The occurrence of homolog proteins was based on BLASTp (NCBI) search against non-redundant protein sequences with the output limited to 5000 targets; the bacterial orders with ≥200 hits are listed in brackets in decreasing order (see corresponding SI Excel files for search parameters)

***** The paralogs were identified based on the BLASTp (NCBI) search against the *N. terpenica* IFM0406 protein sequences with cut-off parameters: 50 bits, 70% query coverage, 20% seq identity (see also Tabs. S5A-E)

Please note that for compatibility with the diagrams showing Bra-BGC, the proteins listed in this table are shown in the reversed order of their appearance in the N. terpenica IFM0406 genome.

Table S5A-E. (see in SI Excel file).

Table S6. (see in SI Excel file).

Table S7. Bioinformatic tools

Name	Internet address and description	Reference
READemption	https://reademption.readthedocs.io/en/latest/#	(Förstner et al. 2014)
TSSpredator	https://tsspredator20-rtd.readthedocs.io/en/latest/index.html	(Dugar et al. 2013)
SMART	http://smart.embl-heidelberg.de/	(Schultz et al. 1998; Letunic and Bork 2018)
ProtParam	https://web.expasy.org/protparam/	(Gasteiger et al. 2005)
blastp	https://www.ncbi.nlm.nih.gov/	
MEME	http://meme-suite.org/tools/meme	(Bailey et al. 2009)
WebLogo	https://weblogo.berkeley.edu/	(Crooks et al. 2004)

Supplementary Materials and Methods

DNA constructs and strains

pET-21a(+) derivatives for expression and purification of recombinant proteins.

To obtain pET-21a(+)kstR, pET-21a(+)sdpR, pET-21a(+)bra12, and pET-21a(+)ompR plasmids the corresponding *kstR*, *sdpR*, *bra12*, and *ompR* genes were PCR amplified using primers listed in Table S2 and bcaAB01 fosmid as a template. Subsequently, the PCR products were purified using commercial silica-based column kits and digested using NdeI and XhoI restriction enzymes, except for bra12 which was digested with NdeI and SalI. After thermal inactivation, the restriction fragments were ligated with previously digested NdeI-XhoI pET-21a(+) vector. Fidelity of the cloned genes was verified by sequencing.

pIJ10257_sdpR

The *sdpR* gene was PCR amplified using primers listed in Table S2 and bcAB01 fosmid as a template. The resulting product was restriction digested, and cloned into the pIJ10257 vector using NdeI and XhoI restriction sites to give pIJ10257_sdpR plasmid.

pUWL201HX and *pUWL201HX_SdpR_MK*

In the first stage the pUWL201HX vector, comprising a hygromycin resistance cassette, was constructed. The plasmid was obtained by cloning the PCR-amplified hygromycin cassette into XbaI restriction site in the original pUWL201. The cassette was amplified using the primers listed in Table S2 and pIJ10700 plasmid as a DNA template. The orientation of the cassette was later confirmed by restriction digestion. The pUWL201HX contains the hygromycin cassette in the 5' – *oriT-hph* – 3' (*hph* – hygromycin B phosphotransferase)orientation downstream of the *ermE* promoter. In the subsequent step, the PCR-amplified *sdpR* gene and *ermE** promoter derived from pIJ10257 were cloned into Acc65I-PstI digested pUWL201HX using SLIC method (Li and Elledge 2007) with modifications described previously for pET-21a(+)lysRNt construction (Wolański et al. 2021). The *sdpR* and *ermE*p* fragments were amplified using the primers listed in Table S2 and bcaAB01 and pIJ10257 as templates, respectively. The obtained construct pUWL201HX_SdpR_MK was verified using restriction digestion and sequencing.

pFLUX vectors for luminescence assays

To obtain pFLUX-bra0p, pFLUX-bra1p, pFLUX-bra12p, and pFLUX-sdpRp plasmids the corresponding *bra0*, *bra1*, *bra12*, and *sdpR* promoter regions (approximately 500 bps gene upstream) were PCR amplified using primers listed in Table S2, and bcaAB01 fosmid as a template. Subsequently, the PCR products were purified using silica-based columns and digested using Acc65I and Eco32I (for bra0p, and bra1p fragments) or NdeI and Eco32I (for bra12p, and sdpRp fragments) restriction enzymes. After thermal inactivation, the restriction fragments were ligated with previously digested pFLUX vector digested with compatible enzymes. Fidelity of the cloned genes was verified by sequencing.

bcaAB01_ΔsdpR and *bcaAB01_Δbra12*

Briefly, for gene deletions on the bcaAB01 fosmid, the PCR-targeting method was used as described earlier (Gust et al. 2003). To replace the gene sequences with the antibiotic resistance cassettes the primers comprising specific restriction sites and 39-nt extensions homologues to the gene sequences were used. In the first stage, disruption cassettes containing an apramycin resistance gene were PCR amplified using the primers listed in Table S2, and a pIJ773 plasmid as a template. Next, the generated PCR products were used to introduce *sdpR* or *bra12* gene replacements on the bcaAB01 fosmid using recombination in *E. coli* BW25113/pIJ790 strain harboring the fosmid, as

previously described (Gust et al. 2003). In the subsequent stages, the disruption cassettes were excised out of the mutated fosmids using PacI restriction enzyme, and the fosmids were re-ligated using T4 ligase to generate SCAR. The correctness of the mutated fosmids was confirmed using various restriction enzymes.

Generation of Escherichia coli, Streptomyces, and Amycolatopsis strain derivatives

Chemically competent *E. coli* DH5α were prepared and transformed according to commonly available protocols. The competent cells of *E. coli* BW/pIJ790 and ET12567/pUZ8002 strains were prepared according to the PCR-targeting protocol (Gust et al. 2003).

For the introduction of DNA constructs into *A. japonicum* strains the intergeneric conjugation procedure was used, as previously described (Schwarz et al. 2018). For the conjugation of the *Streptomyces* strains the standard protocol was followed (Kieser et al. 2000).

Protein expression and purification

Bra12-Hisx6 purification

For the purification of Bra12-Hisx6 protein, the *E. coli* Rosetta 2(DE3) strain (Merck) was transformed with the pET-21a(+)bra12 vector. Preparation of expression culture (1.6-litre) was conducted as described previously (Wolański et al. 2021). For protein extraction the obtained cell pellet was resuspended in 75 mL of lysis buffer A (50 mM NaH_2PO_4, 300 mM NaCl, 10 mM imidazole, pH 8.0) (5 mL/g of cell paste) supplemented with protease inhibitors (A32965, ThermoFisher Scientific) and universal nuclease (88702, ThermoFisher Scientific), followed by disruption performed using sonication (Sonics) (5s ON/ 5s OFF, amplitude 40-50%, 10 minutes sonication time). The cell lysate was then clarified by centrifugation (45 min., 37 000 x g, 4°C), and mixed with chromatography resin – 2 mL (batch volume) of His-Select® Nickel Affinity Gel (P6611, Merck) previously equilibrated in A buffer – followed by overnight incubation at 8°C on rocking platform. Next, the resin was applied onto the gravity column and washed with buffer A supplemented with imidazole (20 and 40 mM final concentration – 15 and 10-bed volumes, respectively). The Bra12-Hisx6 protein was eluted from the resin using buffer A supplemented with 100 and 250 mM imidazole (final concentration) (4-bed volumes for each elution buffer). The elution fractions (250-350 μL) were collected manually and analyzed using sodium dodecyl sulfate-polyacrylamide gel electrophoresis (SDS-PAGE). Similar fractions were then pooled together and aliquoted. The protein samples were flash-frozen with liquid nitrogen and stored at -80°C.

SdpR purification

The SdpR-Hisx6 recombinant protein was overproduced using the *E. coli* BL21(DE3) strain transformed with the pET-21a(+)sdpR plasmid. The overexpression culture (total volume of 250 ml in LB medium) was prepared similarly to the procedure described previously in the $CabRHis_6$ purification protocol (Wolański et al. 2016). The purification of SdpR-Hisx6 was performed using His-Select® Nickel Affinity Gel as described above for Bra12-Hisx6.

KstR-Hisx6 purification

The *E. coli* Rosetta 2(DE3) strain (Merck) transformed with the pET-21a(+)kstR was used to overproduce the KstR-Hisx6 recombinant protein. The KstR-Hisx6 was purified from a 1.6-litre expression culture using the protocol previously described for the LysRNtHis6 protein (Wolański et al. 2021).

OmpR-Hisx6 purification

The *E. coli* Rosetta 2(DE3) strain (Merck) transformed with the pET-21a(+)ompR was used to overproduce the OmpR-Hisx6 recombinant protein. The OmpR-Hisx6 was purified from a 1.6-litre expression culture using the protocol previously described for the LysRNtHis6 protein (Wolański et al. 2021).

Protein-DNA interactions

EMSA assays with digested fosmids

The interaction studies between purified recombinant proteins and restriction-digested BcaAB01 fosmid were performed similarly to those previously described for MBPCabA protein (Wolański et al. 2016). Briefly, the BcaAB01 fosmid DNA was digested using a corresponding restriction enzyme followed by heat-inactivation of the enzyme. The binding reaction was assembled by mixing 2 μL of the recombinant protein, diluted in the protein purification buffer (lysis buffer A with imidazole concentration adjusted to 100 mM), and 18 μL of the reaction mixture containing 750 ng of digested fosmid DNA diluted in 1× phosphate-buffered saline (PBS; 137 mM NaCl, 2.7 mM, KCl, 10 mM Na_2HPO_4, 1.8 mM KH_2PO_4) supplemented with BSA and glycerol (5 μg/μL and 5%, final concentrations, respectively). After a 30-minute incubation at room temperature (~25 °C), the samples were transferred to ice and immediately loaded into 1% agarose gels prepared in 0.5× Tris borate-EDTA (TBE) buffer. The gel was then resolved at 20 V overnight at room temperature, in the 0.5× TBE as a running buffer. The next day, the gels were bathed in ethidium bromide solution (0.5 μg/mL) and DNA was visualized with UV light using the ChemiDoc MP system (Bio-Rad).

EMSA assays with PCR-amplified DNA fragments

EMSAs with non-radiolabeled DNA fragments

The protein-DNA interactions were carried out as described previously (Wolanski et al. 2011; Wolański et al. 2016), with minor changes. Briefly, the purified proteins were incubated with 25–50 ng of PCR-amplified non-labeled DNA fragments for 30 min (~25 °C) in 1× PBS supplemented with 5 % glycerol and 5 μg/μL bovine serum albumin (BSA). In case more than one non-labeled DNA fragment was used in the assay (see Fig. 2A and S6A), all fragments were delivered in the same amounts (50 ng per reaction) apart from internal negative control (33140p) of which 25 ng per reaction was used due to shorter length of the fragment. After incubation, the samples were transferred to ice and immediately run onto 4–5% polyacrylamide gels in 0.25× TBE for 3–4 h (5–10 V/cm) at 4–6°C. Before loading the samples, the gels were pre-run for 30 minutes at 100 V 4-8°C. After the run staining with ethidium bromide was used to visualize the DNA using the ChemiDoc MP apparatus (Bio-Rad).

EMSAs with radiolabeled DNA fragments

The protein-DNA interactions were carried out as described previously (Zawilak-Pawlik et al. 2005; Wolański et al. 2021), with the following modifications. Briefly, the purified recombinant proteins were incubated with 50 cps ^{32}P-radiolabeled DNA (^{32}P) (~ 4 fmol) in the reaction buffer for 30 minutes at 25°C. The binding reactions were conducted in 20 µl in 1× PBS supplemented with 5 % glycerol and 5 µg/µL BSA, and non-specific competitor poly(dI-dC)·(dI-dC) (3.75 ng/µL). In the competition assays with non-labeled DNA fragments, the "cold" DNAs were added to the reaction mixtures before the corresponding regulatory proteins. In case a restriction digestion of radiolabeled fragments was required the restriction reaction was conducted on radiolabeled DNA fragments, then after thermal inactivation of the enzymes the fragments were incubated with the proteins as described above. After incubation, the samples were resolved in 4–6% polyacrylamide gels. Upon the electrophoresis, the gels were dried onto a single sheet of Whatmann 3MM paper using a vacuum gel dryer. The gels were subsequently incubated overnight with a Storage Phosphor Screen (GE Healthcare) and analyzed using an autoradiography reader (Typhoon 8600 Variable Mode Imager, GE Healthcare), and dedicated software (Image Quant, GE Healthcare).

DNase I footprinting assay

The footprinting experiments were performed similarly to a previously described method (Majka et al. 1999; Wolanski et al. 2011). Briefly, ~10 fmols of DNA fragment, PCR amplified using 5′-end radiolabelled oligonucleotide, was incubated with different amounts of a recombinant protein in

1× reaction buffer (the same as used for EMSAs) at 25 °C for 30 min followed by DNase I treatment. Upon stopping the samples were applied on an 8 % polyacrylamide-urea sequencing gel and resolved. The gel was analyzed using the Typhoon 8600 Variable Mode Imager and Image Quant software.

RT-qPCR

For gene expression analyses using qPCR the *A. japonicum* strains were cultivated as described previously (Schwarz et al. 2018). The sample collection at 72 hours of growth and subsequent steps including total RNA isolation, cDNA synthesis, and qPCR analyses were performed according to the procedure described in our previous report for *S. venezuelae* (Płachetka et al. 2021). The experiments were conducted in three biological replicates for each strain in the qPCR experiment. The negative-control reactions (no reverse transcriptase added) were performed to confirm the absence of chromosomal DNA contamination. The primers used in the study are listed in Table S2.

Internet links to sequence resources

This genome sequence has been deposited in EMBL/GenBank under the accession number NZLWGR00000000.1 (https://ftp.ncbi.nlm.nih.gov/genomes/all/GCF/001/625/105/GCF_001625105.1_ASM162510v1/); the brasilicardin gene cluster sequence is available under GenBank accession number MT247069 (direct link: https://www.ncbi.nlm.nih.gov/nuccore/MT247069). To get direct access to whole-genome nucleotide and protein sequences use the following genome annotation link: https://www.ncbi.nlm.nih.gov/datasets/genome/GCF_001625105.1/

Raw sequencing data are available via Gene Expression Omnibus under accession number GSE271981 (https://www.ncbi.nlm.nih.gov/geo/query/acc.cgi?acc=GSE271981).

Supplementary references

Bailey TL, Boden M, Buske FA, Frith M, Grant CE, Clementi L, Ren J, Li WW, Noble WS (2009) MEME Suite: Tools for motif discovery and searching. Nucleic Acids Res 37:W202–W208. https://doi.org/10.1093/nar/gkp335

Craney A, Hohenauer T, Xu Y, Navani NK, Li Y, Nodwell J (2007) A synthetic *luxCDABE* gene cluster optimized for expression in high-GC bacteria. Nucleic Acids Res 35:e46. https://doi.org/10.1093/nar/gkm086

Crooks GE, Hon G, Chandonia JM, Brenner SE (2004) WebLogo: A sequence logo generator. Genome Res 14:1188–1190. https://doi.org/10.1101/gr.849004

Doumith M, Weingarten P, Wehmeier UF, Salah-Bey K, Benhamou B, Capdevila C, Michel J-M, Piepersberg W, Raynal M-C (2000) Analysis of genes involved in 6-deoxyhexose biosynthesis and transfer in *Saccharopolyspora erythraea* . Mol Gen Genet 264:477–485. https://doi.org/10.1007/s004380000329

Dugar G, Herbig A, Förstner KU, Heidrich N, Reinhardt R, Nieselt K, Sharma CM (2013) High-resolution transcriptome maps reveal strain-specific regulatory features of multiple *Campylobacter jejuni* isolates. PLoS Genet 9:e1003495. https://doi.org/10.1371/journal.pgen.1003495

Förstner KU, Vogel J, Sharma CM (2014) READemption-a tool for the computational analysis of deep-sequencing-based transcriptome data. Bioinformatics 30:3421–3423. https://doi.org/10.1093/bioinformatics/btu533

Gasteiger E, Hoogland C, Gattiker A, Duvaud S, Wilkins MR, Appel RD, Bairoch A (2005) Protein Identification and Analysis Tools on the ExPASy Server. In: The Proteomics Protocols Handbook. Humana Press, pp 571–607

Gomez-Escribano JP, Bibb MJ (2011) Engineering *Streptomyces coelicolor* for heterologous expression of secondary metabolite gene clusters. Microb Biotechnol 4:207–215. https://doi.org/10.1111/j.1751-7915.2010.00219.x

Gust B, Challis GL, Fowler K, Kieser T, Chater KF (2003) PCR-targeted *Streptomyces* gene replacement identifies a protein domain needed for biosynthesis of the sesquiterpene soil odor geosmin. Proc Natl Acad Sci U S A 100:1541–6. https://doi.org/10.1073/pnas.0337542100

Hong HJ, Hutchings MI, Hill LM, Buttner MJ (2005) The role of the novel fem protein VanK in vancomycin resistance in *Streptomyces coelicolor* . J Biol Chem 280:13055–13061. https://doi.org/10.1074/jbc.M413801200

Kieser T, Bibb MJ, Chater KF, Butter MJ, Hopwood DA (2000) Practical *Streptomyces* Genetics: A Laboratory Manual. John Innes Foundation, Norwich, United Kingdom

Letunic I, Bork P (2018) 20 years of the SMART protein domain annotation resource. Nucleic Acids Res 46:D493–D496. https://doi.org/10.1093/nar/gkx922

Li MZ, Elledge SJ (2007) Harnessing homologous recombination *in vitro* to generate recombinant DNA via SLIC. Nat Methods 4:251–256. https://doi.org/10.1038/nmeth1010

MacNeil DJ, Gewain KM, Ruby CL, Dezeny G, Gibbons PH, MacNeil T (1992) Analysis of *Streptomyces avermitilis* genes required for avermectin biosynthesis utilizing a novel integration vector. Gene 111:61–68. https://doi.org/10.1016/0378-1119(92)90603-M

Majka J, Jakimowicz D, Messer W, Schrempf H, Lisowski M, Zakrzewska-Czerwińska J (1999) Interactions of the *Streptomyces lividans* initiator protein DnaA with its target. Eur J Biochem 260:325–335. https://doi.org/10.1046/j.1432-1327.1999.00168.x

Nishikiori T, Okuyama A, Naganawa H, Takita T, Hamada M, Takeuchi T, Aoyagi T, Umezawa H (1984) Production by actinomycetes of (s,s)-n,;n'-ethylenediamine-disuccinic acid, an inhibitor of phospholipase c. J Antibiot 37:426–427. https://doi.org/10.7164/antibiotics.37.426

Paget MSB, Chamberlin L, Atrih A, Foster SJ, Buttner MJ (1999) Evidence that the extracytoplasmic function sigma factor σ^E is required for normal cell wall structure in *Streptomyces coelicolor* A3(2). J Bacteriol 181:204–211. https://doi.org/10.1128/jb.181.1.204-211.1999

Płachetka M, Krawiec M, Zakrzewska-Czerwińska J, Wolański M (2021) AdpA Positively Regulates Morphological Differentiation and Chloramphenicol Biosynthesis in *Streptomyces venezuelae*. Microbiol Spectr 9. https://doi.org/10.1128/spectrum.01981-21

Schultz J, Milpetz F, Bork P, Ponting CP (1998) SMART, a simple modular architecture research tool: Identification of signaling domains. Proc Natl Acad Sci U S A 95:5857–5864. https://doi.org/10.1073/pnas.95.11.5857

Schwarz PN, Buchmann A, Roller L, Kulik A, Gross H, Wohlleben W, Stegmann E (2018) The immunosuppressant brasilicardin: determination of the biosynthetic gene cluster in the heterologous host *Amycolatopsis japonicum* . Biotechnol J 13:1–12. https://doi.org/10.1002/biot.201700527

Wolanski M, Donczew R, Kois-Ostrowska A, Masiewicz P, Jakimowicz D, Zakrzewska-Czerwinska J (2011) The level of AdpA directly affects expression of developmental genes in *Streptomyces coelicolor* . J Bacteriol 193:6358–65. https://doi.org/10.1128/JB.05734-11

Wolański M, Krawiec M, Schwarz PN, Stegmann E, Wohlleben W, Buchmann A, Gross H, Eitel M, Koch P, Botas A, Méndez C, Núñez LE, Morís F, Cortés J, Zakrzewska-Czerwińska J (2021) A novel LysR-type regulator negatively affects biosynthesis of the immunosuppressant brasilicardin. Eng Life Sci 21:4–18. https://doi.org/10.1002/elsc.202000038

Wolański M, Łebkowski T, Kois-Ostrowska A, Zettler J, Apel AK, Jakimowicz D, Zakrzewska-Czerwińska J (2016) Two transcription factors, CabA and CabR, are independently involved in multilevel regulation of the biosynthetic gene cluster encoding the novel aminocoumarin, cacibiocin. Appl Microbiol Biotechnol 100:3147–3164. https://doi.org/10.1007/s00253-015-7196-7

Zawilak-Pawlik A, Kois A, Majka J, Jakimowicz D, Smulczyk-Krawczyszyn A, Messer W, Zakrzewska-Czerwińska J (2005) Architecture of bacterial replication initiation complexes: orisomes from four unrelated bacteria. Biochem J 389:471–481. https://doi.org/10.1042/BJ20050143

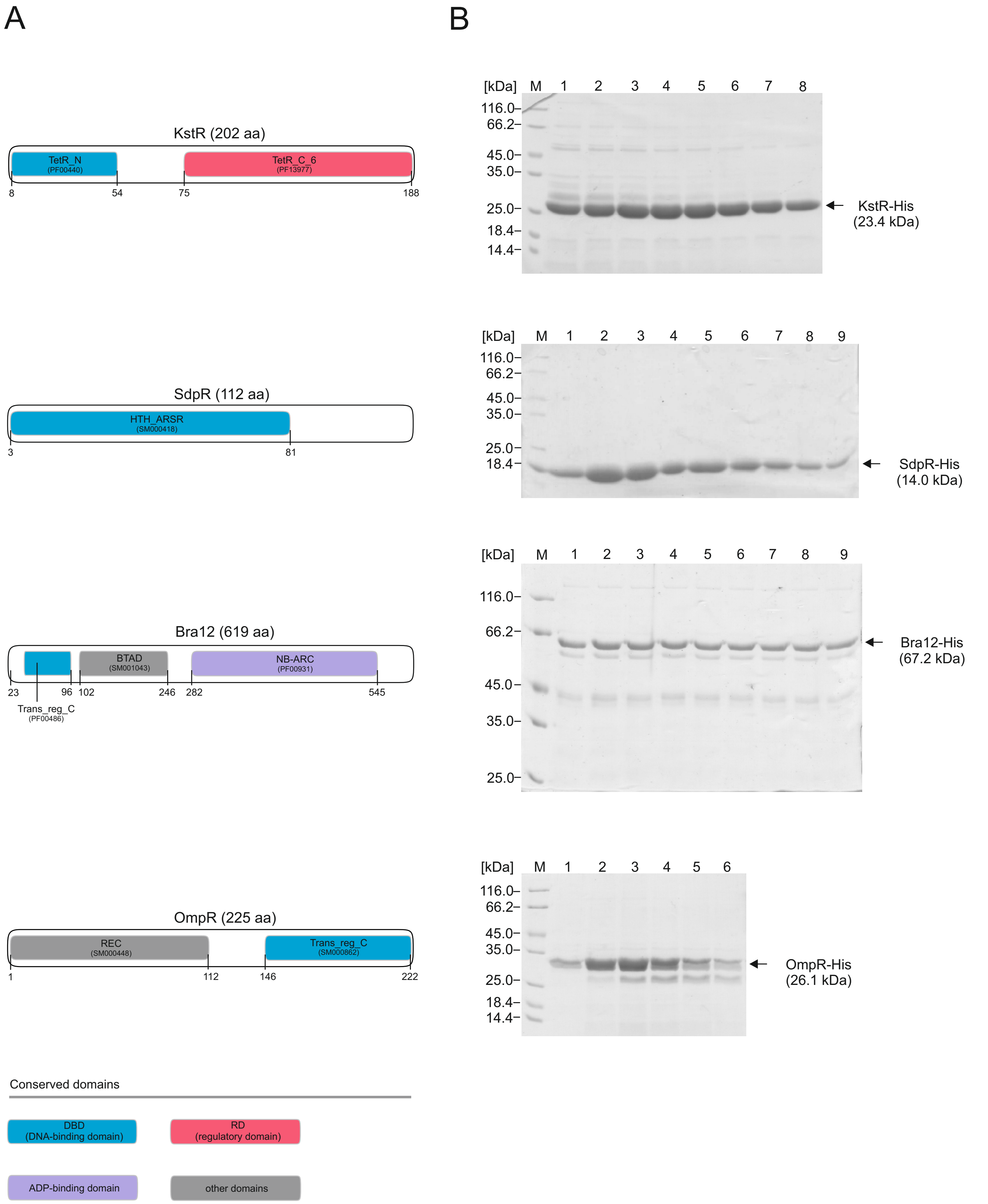
A
B
KstR (202 aa)
TetR_N
(PF00440)
TetR_C_6
(PF13977)
8
54
75
188
SdpR (112 aa)
HTH_ARSR
(SM000418)
3
81
Bra12 (619 aa)
BTAD
(SM001043)
NB-ARC
(PF00931)
23
96
102
246
282
545
Trans_reg_C
(PF00486)
OmpR (225 aa)
REC
(SM000448)
Trans_reg_C
(SM000862)
1
112
146
222
Conserved domains
DBD
(DNA-binding domain)
RD
(regulatory domain)
ADP-binding domain
other domains
[kDa] M 1 2 3 4 5 6 7 8
116.0
66.2
45.0
35.0
25.0
18.4
14.4
KstR-His
(23.4 kDa)
[kDa] M 1 2 3 4 5 6 7 8 9
SdpR-His
(14.0 kDa)
Bra12-His
(67.2 kDa)
[kDa] M 1 2 3 4 5 6
OmpR-His
(26.1 kDa)

Fig. S1

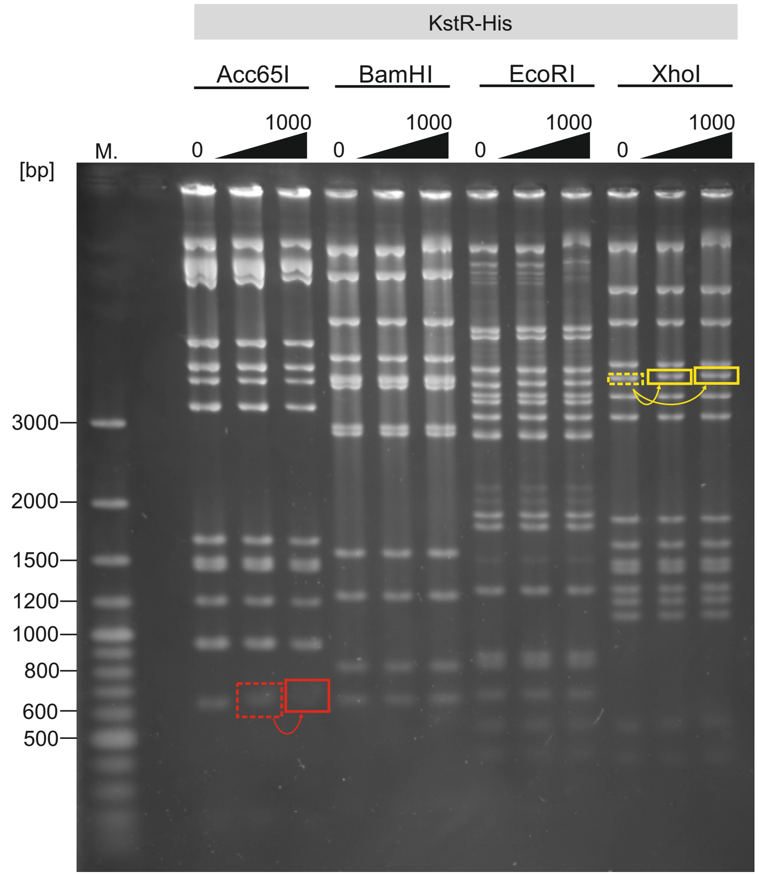
KstR-His
Acc65I
BamHI
EcoRI
XhoI
0
1000
M.
[bp]
3000
2000
1500
1200
1000
800
600
500

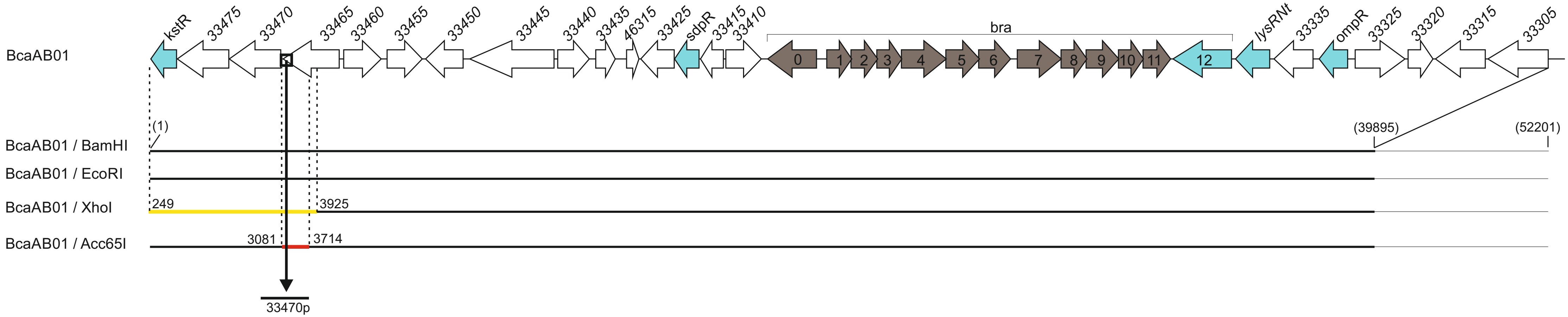
BcaAB01
kstR
33475
33470
33465
33460
33455
33450
33445
33440
33435
46315
33425
sdpR
33415
33410
bra
0
1
2
3
4
5
6
7
8
9
10
11
12
lysRNt
33335
ompR
33325
33320
33315
33305
(1)
(39895)
(52201)
BcaAB01 / BamHI
BcaAB01 / EcoRI
BcaAB01 / XhoI
249
3925
BcaAB01 / Acc65I
3081
3714
33470p

Fig. S2

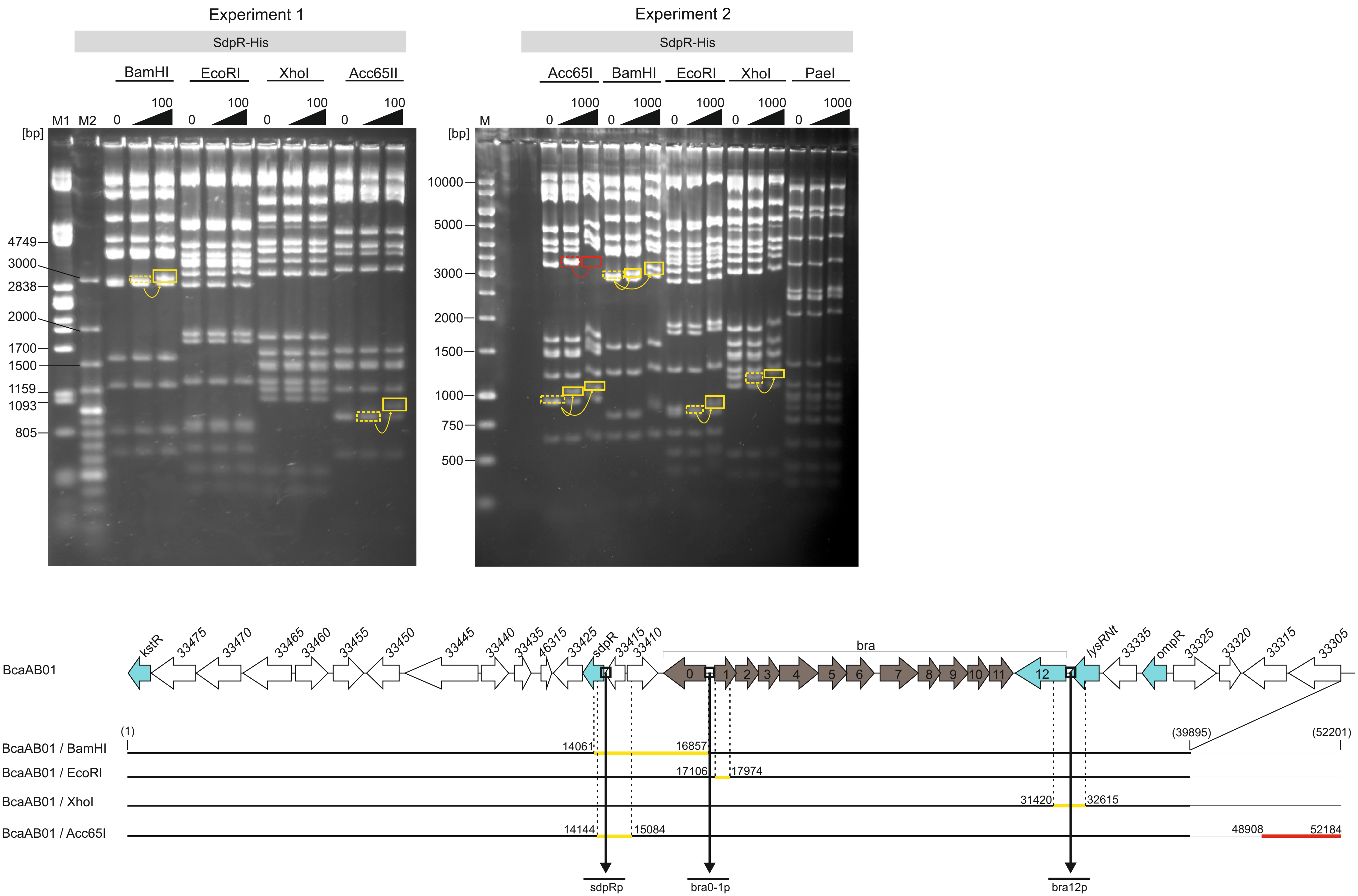

Experiment 1
SdpR-His
BamHI
EcoRI
XhoI
Acc65II
M1 M2
0 100
[bp]
4749
3000
2838
2000
1700
1500
1159
1093
805
Experiment 2
SdpR-His
Acc65I
BamHI
EcoRI
XhoI
PaeI
M
0 1000
[bp]
10000
5000
3000
2000
1500
1000
750
500
BcaAB01
kstR
33475
33470
33465
33460
33455
33450
33445
33440
33435
46315
33425
sdpR
33415
33410
bra
0 1 2 3 4 5 6 7 8 9 10 11 12
lysRNt
33335
ompR
33325
33320
33315
33305
(1)
(39895)
(52201)
BcaAB01 / BamHI
14061
16857
BcaAB01 / EcoRI
17106
17974
BcaAB01 / XhoI
31420
32615
BcaAB01 / Acc65I
14144
15084
48908
52184
sdpRp
bra0-1p
bra12p

Fig. S3

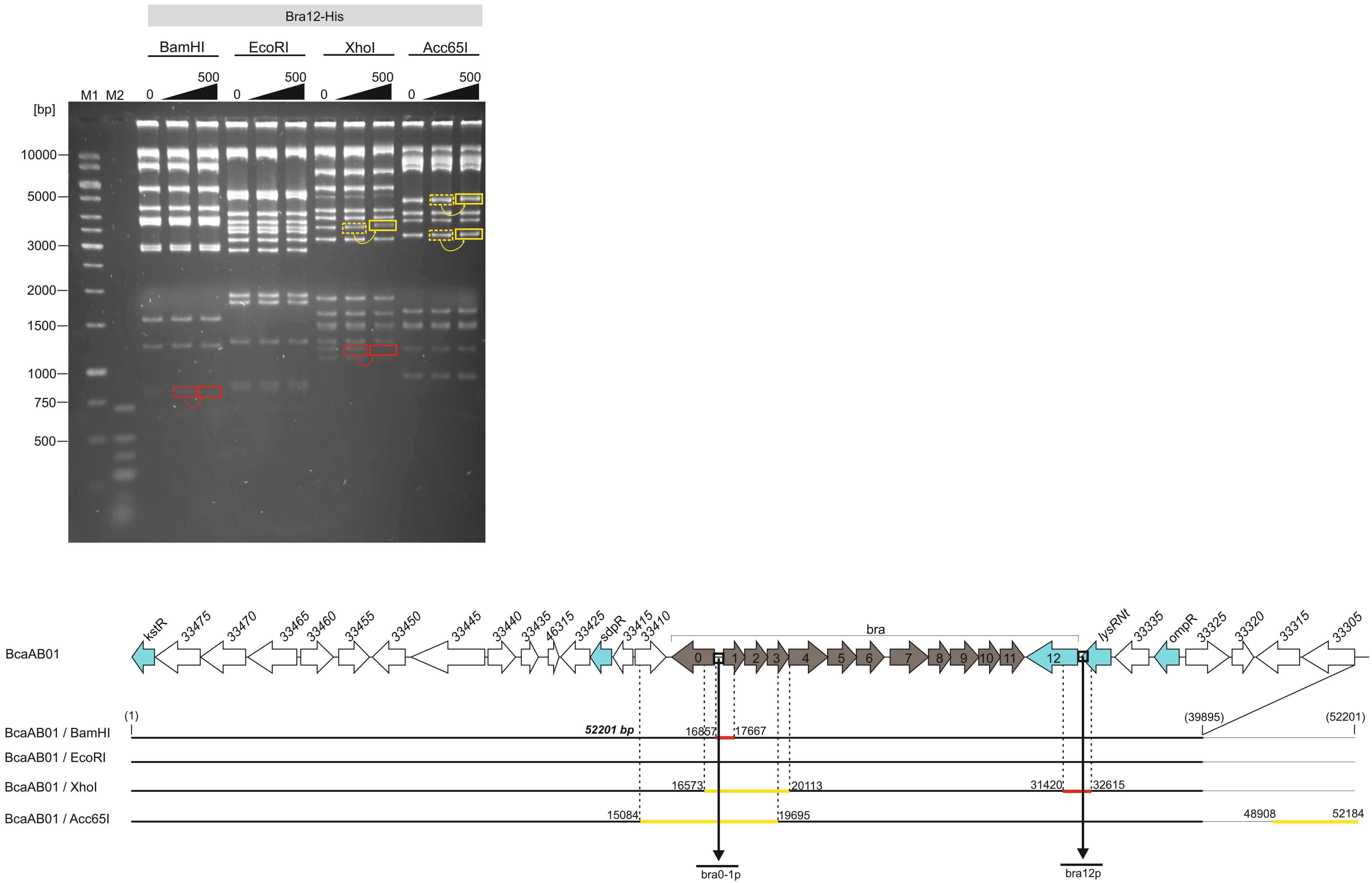
Bra12-His
BamHI
EcoRI
XhoI
Acc65I
0
500
M1
M2
[bp]
10000
5000
3000
2000
1500
1000
750
500
BcaAB01
kstR
33475
33470
33465
33460
33455
33450
33445
33440
33435
46315
33425
sdpR
33415
33410
bra
0
1
2
3
4
5
6
7
8
9
10
11
12
lysRNt
33335
ompR
33325
33320
33315
33305
(1)
BcaAB01 / BamHI
52201 bp
16857
17667
(39895)
(52201)
BcaAB01 / EcoRI
BcaAB01 / XhoI
16573
20113
31420
32615
BcaAB01 / Acc65I
15084
19695
48908
52184
bra0-1p
bra12p

Fig. S4

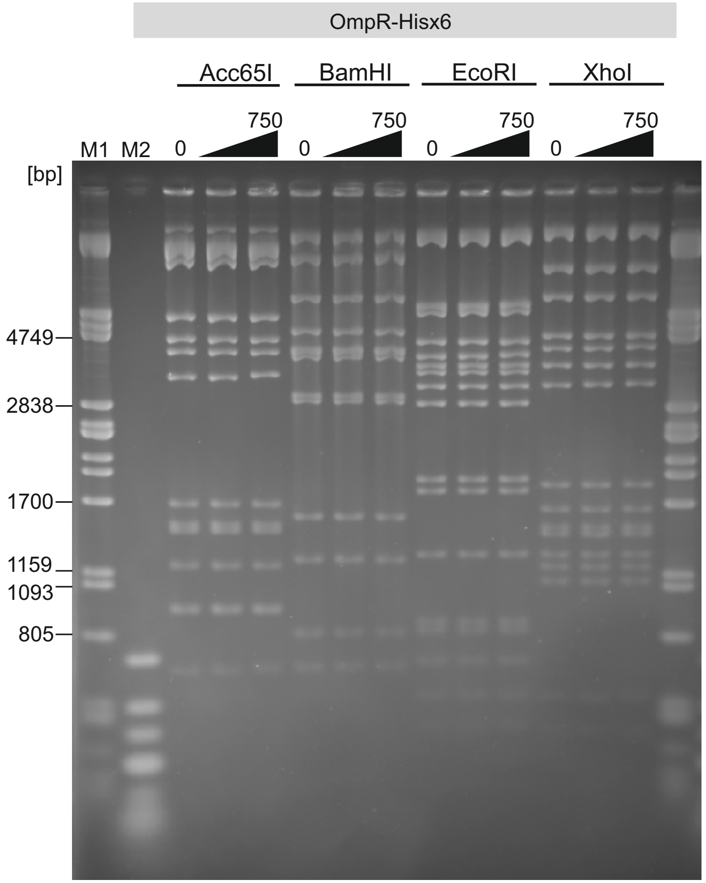
OmpR-Hisx6
Acc65I
BamHI
EcoRI
XhoI
750
M1
M2
0
[bp]
4749
2838
1700
1159
1093
805

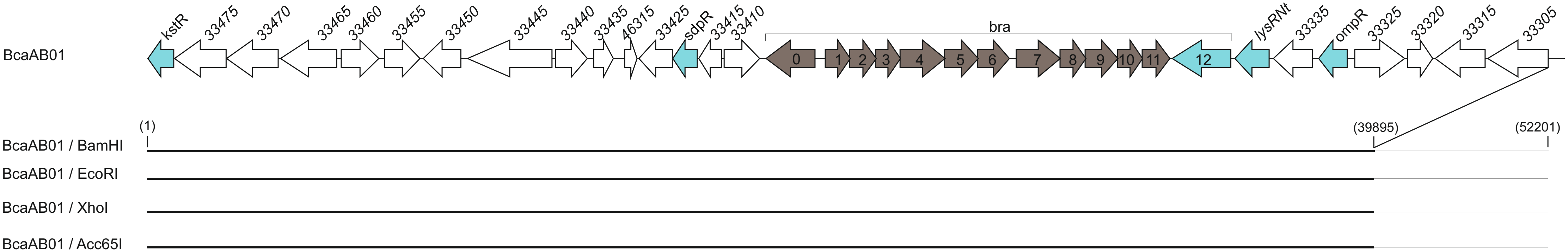
BcaAB01
kstR
33475
33470
33465
33460
33455
33450
33445
33440
33435
46315
33425
sdpR
33415
33410
bra
0
1
2
3
4
5
6
7
8
9
10
11
12
lysRNt
33335
ompR
33325
33320
33315
33305
(1)
(39895)
(52201)
BcaAB01 / BamHI
BcaAB01 / EcoRI
BcaAB01 / XhoI
BcaAB01 / Acc65I

Fig. S5

A

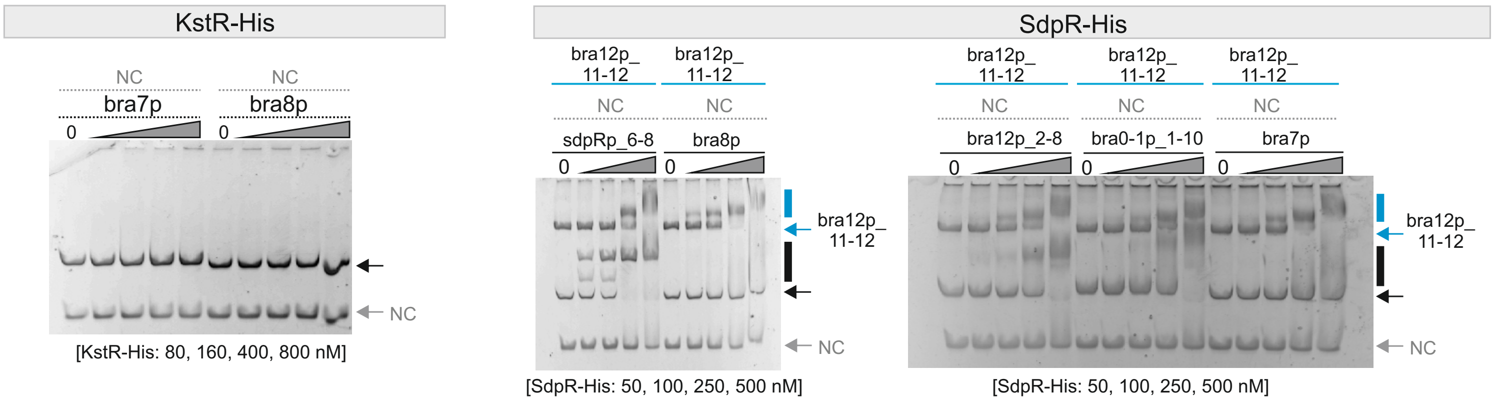
KstR-His
NC
bra7p
NC
bra8p
0
0
NC
[KstR-His: 80, 160, 400, 800 nM]
SdpR-His
bra12p_ 11-12
bra12p_ 11-12
NC
NC
sdpRp_6-8
bra8p
0
0
bra12p_ 11-12
NC
[SdpR-His: 50, 100, 250, 500 nM]
bra12p_ 11-12
bra12p_ 11-12
bra12p_ 11-12
NC
NC
NC
bra12p_2-8
bra0-1p_1-10
bra7p
0
0
0
bra12p_ 11-12
NC
[SdpR-His: 50, 100, 250, 500 nM]

B

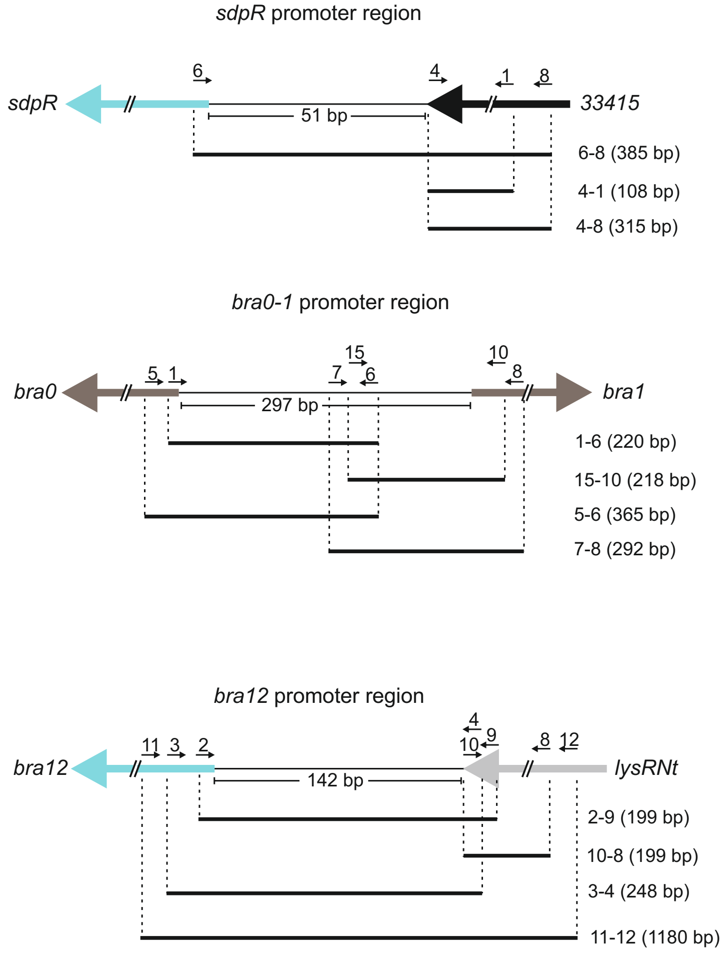
sdpR promoter region
sdpR
6
4
1
8
33415
51 bp
6-8 (385 bp)
4-1 (108 bp)
4-8 (315 bp)
bra0-1 promoter region
bra0
5
1
15
7
6
10
8
bra1
297 bp
1-6 (220 bp)
15-10 (218 bp)
5-6 (365 bp)
7-8 (292 bp)
bra12 promoter region
bra12
11
3
2
4
10
9
8
12
lysRNt
142 bp
2-9 (199 bp)
10-8 (199 bp)
3-4 (248 bp)
11-12 (1180 bp)

Fig. S6

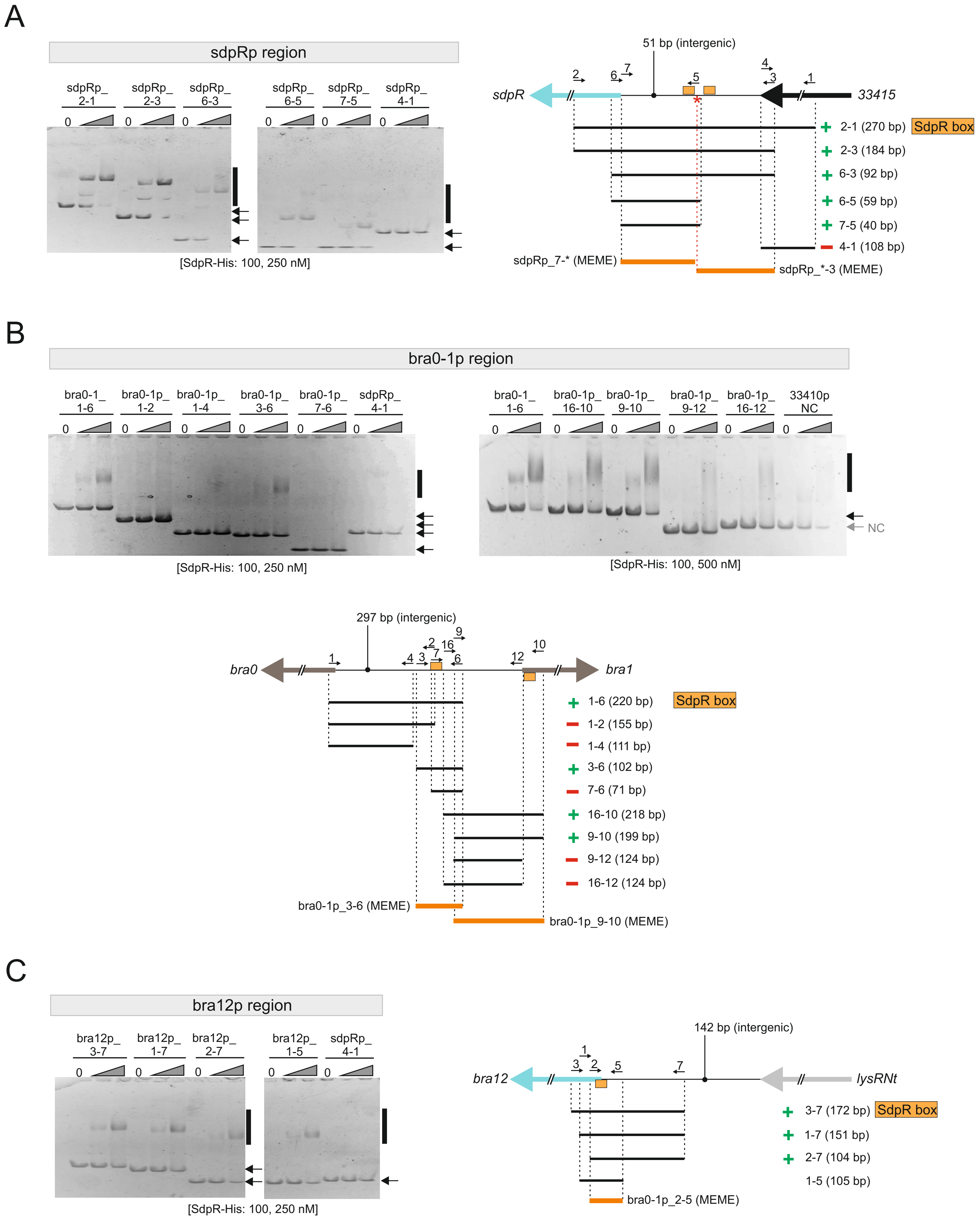
A
sdpRp region
sdpRp_ 2-1
sdpRp_ 2-3
sdpRp_ 6-3
sdpRp_ 6-5
sdpRp_ 7-5
sdpRp_ 4-1
[SdpR-His: 100, 250 nM]
51 bp (intergenic)
sdpR
33415
2-1 (270 bp)
SdpR box
2-3 (184 bp)
6-3 (92 bp)
6-5 (59 bp)
7-5 (40 bp)
4-1 (108 bp)
sdpRp_7-* (MEME)
sdpRp_*-3 (MEME)
B
bra0-1p region
bra0-1_ 1-6
bra0-1p_ 1-2
bra0-1p_ 1-4
bra0-1p_ 3-6
bra0-1p_ 7-6
sdpRp_ 4-1
[SdpR-His: 100, 250 nM]
bra0-1_ 1-6
bra0-1p_ 16-10
bra0-1p_ 9-10
bra0-1p_ 9-12
bra0-1p_ 16-12
33410p NC
NC
[SdpR-His: 100, 500 nM]
297 bp (intergenic)
bra0
bra1
1-6 (220 bp)
SdpR box
1-2 (155 bp)
1-4 (111 bp)
3-6 (102 bp)
7-6 (71 bp)
16-10 (218 bp)
9-10 (199 bp)
9-12 (124 bp)
16-12 (124 bp)
bra0-1p_3-6 (MEME)
bra0-1p_9-10 (MEME)
C
bra12p region
bra12p_ 3-7
bra12p_ 1-7
bra12p_ 2-7
bra12p_ 1-5
sdpRp_ 4-1
[SdpR-His: 100, 250 nM]
142 bp (intergenic)
bra12
lysRNt
3-7 (172 bp)
SdpR box
1-7 (151 bp)
2-7 (104 bp)
1-5 (105 bp)
bra0-1p_2-5 (MEME)

Fig. S7

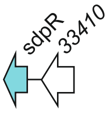

sdpR promoter region

ACGTCGAACGCTACTGT**CAc**AACCGCCAGTGTGCCTCTTTGGTTATATTCCCGTCAAGGCATATAGGGCCG**TCA**GCCCAC
GCCGCCGTCGCGTTCGCGGGTGGCGGCCAGGAGGTCTTCGCGGGCGCGGGCGATGCGAGAGCGGACGGTGCCCACGGGGC
AGCCGCAGACCTCGGCGGCCTCCTGGTAGGACAGGCCCAGGACCTGGGTGAGGATCAGGGCCTCGCGGCGTTCGGGGTCC
AGGCCGTCGAGCAGCATGCGGATCTCGACGAGGTCCTCGAAGGTGCGGCCGGTGCGCTGCACGGCGGCGCGCTGTTCGGC

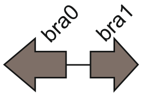

bra0-bra1 promoter region

ACGGTAATGGTCGCAGG**CAT**CGATGACCCCTCCTGGCCGCAACGGGAAGCCGA**c**GGCTGCCATGGTATGTCGCGGATCCG
AATCGCGCAGCCCAACGAGCGGATAAATTTAGGATAAACAATGCTTCCGGACAATAATTTCTGGATAACATCGAAACCGG
TCATATTGCCCGTGATCGGTTTTCCGTGGGATGATCCCGGC**a**TACATTCGGTACGGGGTGGAGGTCGGACGGTCCGGCAA
TTCTGTCGATCATGAATTGGTGAGTTTTTCCGGTGGCGTCACGGCGAAACCGGCGGTTGATAGCGGAGGGTCCGGTG**ATG**
GTGAATTCCGGAGAATG

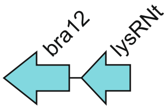

bra12 promoter region

CCCAGCCGAACCAGTGA**CAT**ATAACCTCCCGGATCGAAAACTGGTGCACCAGAAGGCTTCTGGATCATCAGACTCCCCCG
GGTGTG**g**ATAGTCAGTATGTCAAACGATCTCTGTCCGGGATGGTCCTCGGCATAAATTTTGGGTAATACCCTGATAATCC
GG**TCA**CGGCAGCGGCAGGCCGT

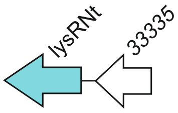

lysRNt promoter region

GCGGTCGAGGACCACTC**CAT**GGCGAAACAACATACCCGGCAGTCGAATCCGTCGTTCCGCAAGAGATTGATCGCGAACAA
TCCGGAGCGG**c**CCACCTATTATCCAGGCGTGCTACCACCCGTTGTCCCGGCGTGCTTTTGGCCGGGACCACCCGCCCGCA
GTG**TCA**GCGGACCGCGGCCGCGA

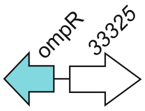

ompR promoter region

TCCACGATCAATAAACG**CAT**CGAGCACACCGCCAATCCGCTAGGTGTATCGGTCTGTCGCGCAATGCCTTCGGCGCACCT
CGACGGGGCGCCCGTGTA**c**GGTCAGATTATGCCCGAGTACCCGCAGTTACGGGAAACCTAGTGCT**ATG**CAGGGGATTTCG
CTCCG

Fig. S8

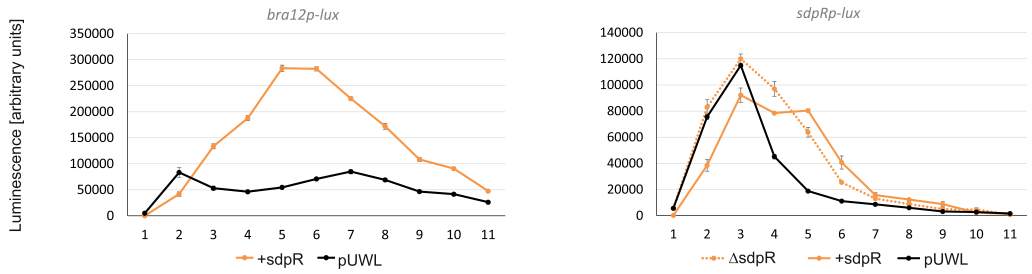
bra12p-lux
sdpRp-lux
Luminescence [arbitrary units]
350000
300000
250000
200000
150000
100000
50000
0
140000
120000
100000
80000
60000
40000
20000
1 2 3 4 5 6 7 8 9 10 11
+sdpR
pUWL
ΔsdpR

Fig. S9

A

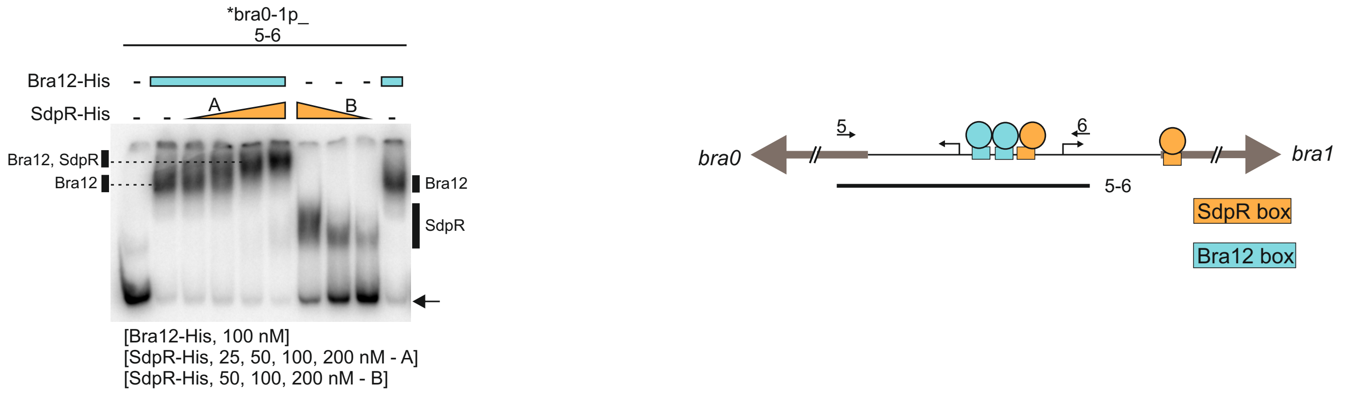

*bra0-1p_
5-6
Bra12-His
SdpR-His
A
B
Bra12, SdpR
Bra12
Bra12
SdpR
[Bra12-His, 100 nM]
[SdpR-His, 25, 50, 100, 200 nM - A]
[SdpR-His, 50, 100, 200 nM - B]
5
6
bra0
bra1
5-6
SdpR box
Bra12 box

B

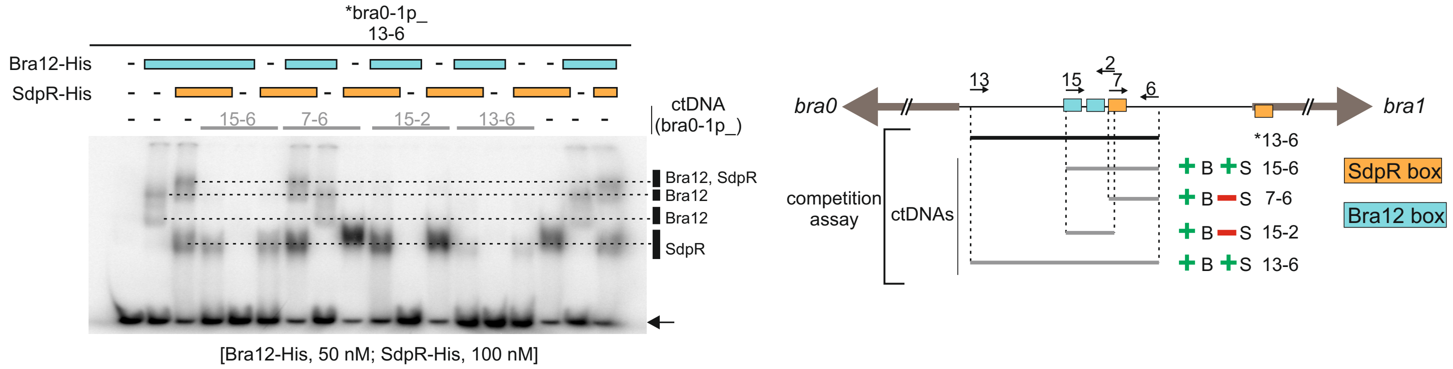

*bra0-1p_
13-6
Bra12-His
SdpR-His
15-6
7-6
15-2
13-6
ctDNA
(bra0-1p_)
Bra12, SdpR
Bra12
Bra12
SdpR
[Bra12-His, 50 nM; SdpR-His, 100 nM]
13
15
2
7
6
bra0
bra1
*13-6
competition
assay
ctDNAs
B S 15-6
B S 7-6
B S 15-2
B S 13-6
SdpR box
Bra12 box

Fig. S10

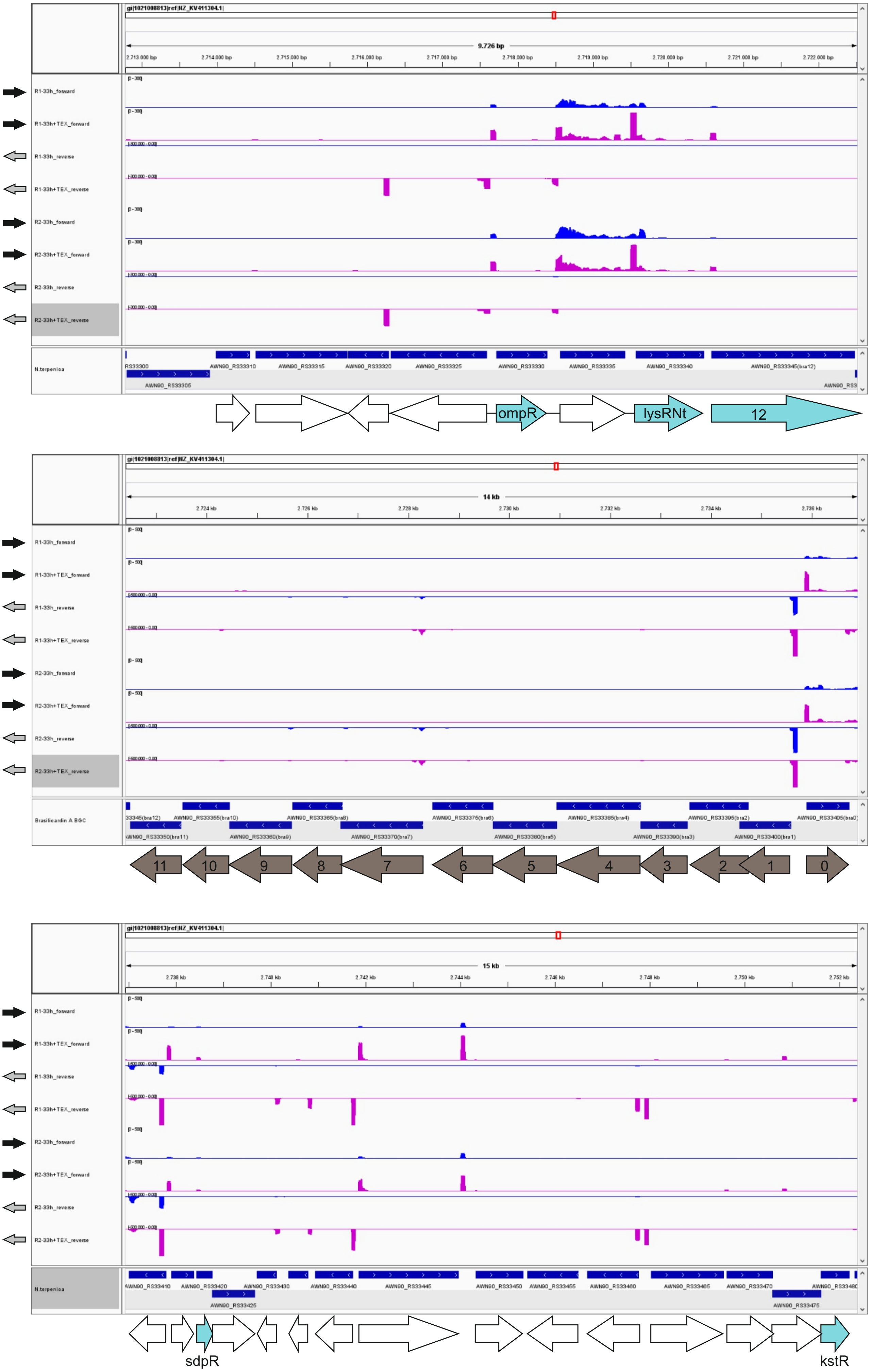
gi|1021008813|ref|NZ_KV411304.1|
9.726 bp
R1-33h_forward
R1-33h+TEX_forward
R1-33h_reverse
R1-33h+TEX_reverse
R2-33h_forward
R2-33h+TEX_forward
R2-33h_reverse
R2-33h+TEX_reverse
N.terpenica
AWN90_RS33305
AWN90_RS33310
AWN90_RS33315
AWN90_RS33320
AWN90_RS33325
AWN90_RS33330
AWN90_RS33335
AWN90_RS33340
AWN90_RS33345(bra12)
ompR
lysRNt
12
14 kb
Brasilicardin A BGC
11
10
9
8
7
6
5
4
3
2
1
0
15 kb
sdpR
kstR

Fig. S11

A

B

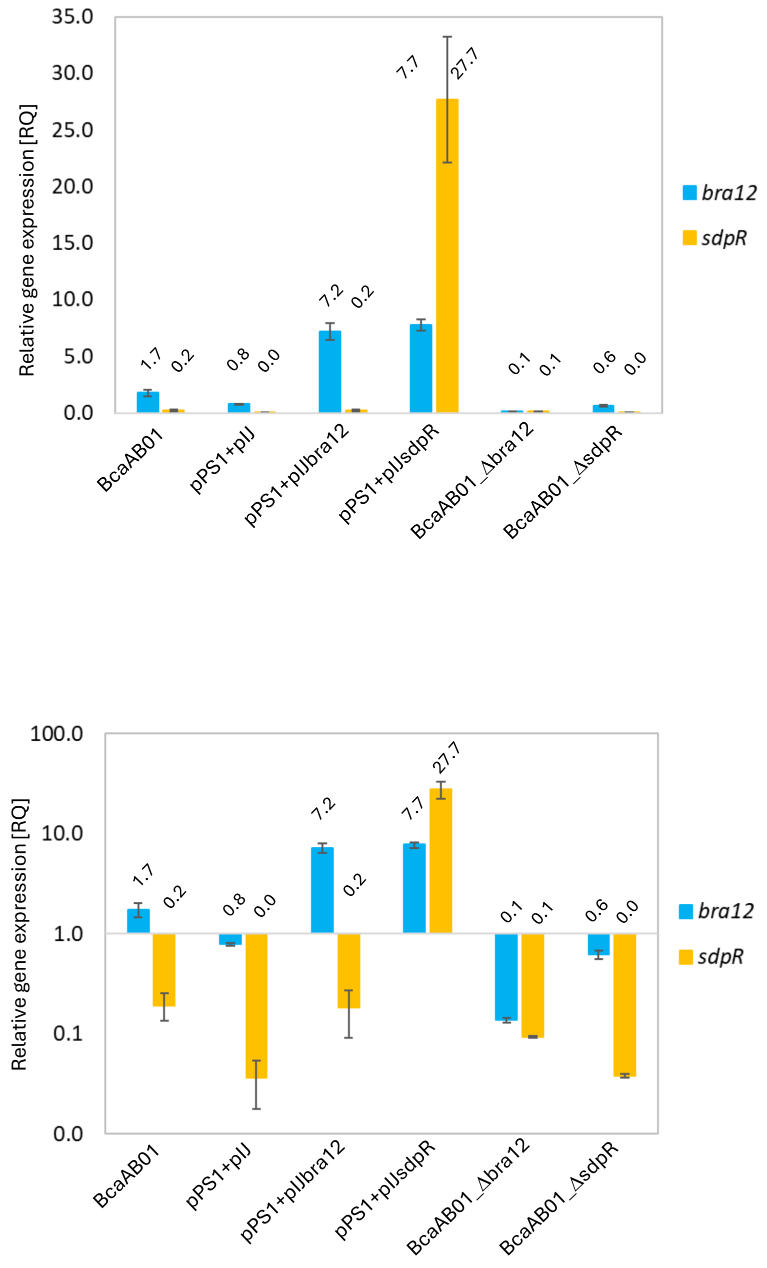

35.0
30.0
25.0
20.0
15.0
10.0
5.0
0.0
Relative gene expression [RQ]
1.7
0.2
0.8
0.0
7.2
0.2
7.7
27.7
0.1
0.1
0.6
0.0
BcaAB01
pPS1+pIJ
pPS1+pIJbra12
pPS1+pIJsdpR
BcaAB01_Δbra12
BcaAB01_ΔsdpR
bra12
sdpR
100.0
10.0
1.0
0.1
0.0

Fig. S12
